# Rapid personalized computational modeling of the wrist

**DOI:** 10.1088/1873-4030/ae8542

**Published:** 2026-08-18

**Authors:** Thor E Andreassen, Taylor P Trentadue, Andrew R Thoreson, Kai-Nan an, Sanjeev Kakar, Kristin D Zhao

**Affiliations:** 1 Assistive and Restorative Technology Laboratory, Mayo Clinic, Rochester, MN, United States of America; 2 Mayo Clinic Medical Scientist Training Program, Mayo Clinic, Rochester, MN, United States of America; 3 Mayo Clinic Graduate Program in Biomedical Engineering and Physiology, Mayo Clinic, Rochester, MN, United States of America; 4 Department of Orthopedic Surgery, Mayo Clinic, Rochester, MN, United States of America; 5 Department of Physical Medicine and Rehabilitation, Mayo Clinic, Rochester, MN, United States of America

**Keywords:** computational modeling and simulation, finite element model, hand, wrist, scapholunate, Monte Carlo analysis, four-dimensional computed tomography

## Abstract

Computational modeling may help to develop new treatments for hand and wrist injuries, but at present, few models exist, as the time and expertise required is considerable. Moreover, most models do not allow for variation of material properties. We have developed an automated workflow combining morphing with algorithmic techniques to create personalized finite element models. Using this workflow, three personalized models were created from our existing four-dimensional computed tomography data. These were then used to demonstrate the usefulness of these models to investigate clinical questions, namely calibration of ligament properties to participant-obtained kinematics, and Monte Carlo analysis of the impacts of ligament injury on joint contact pressure, as an analogue for joint injury that may lead to osteoarthritis. New models can be created in 2 h and individual simulations performed in 45 s. This work enables future patient-specific modeling. Lastly, to encourage reproducibility, we have made the data, models, and code publicly available online.

## 1. Introduction

**Figure 1.**
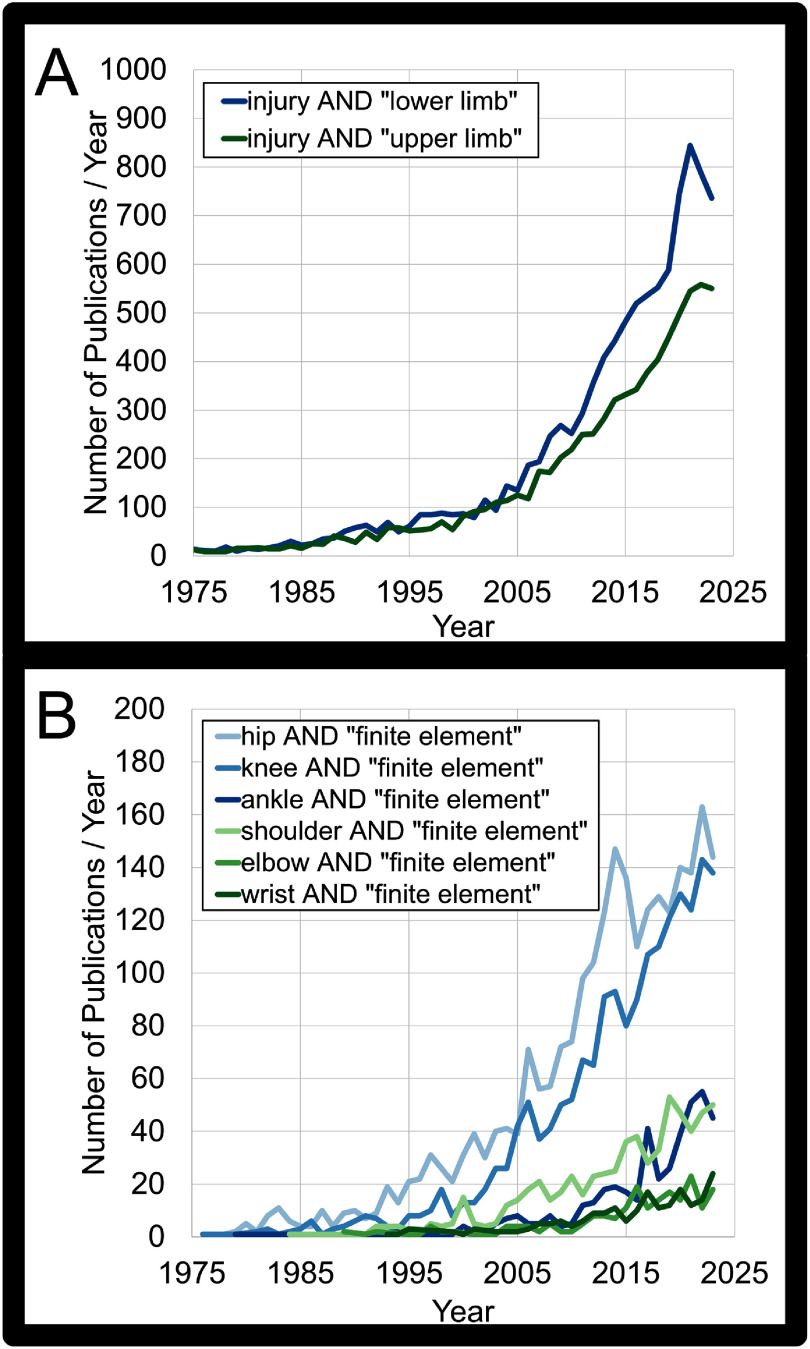
PubMed-indexed articles about lower and upper extremity joints from 1975 to 2024. (A) The number of injury-focused publications is similar between the upper and lower limb. (B) Publications per year focused on ‘finite element’ stratified by joint. The wrist has the least finite element model-based publications of any joint queried. The legend contains the exact search terms used in PubMed.

Despite comparable numbers of publications between upper and lower limb injury research (figure 1(A)), applications of finite element modeling (FEM) predominate in the lower extremity (figure 1(B)). Of the major appendicular joints, the wrist lags in FEM-based studies (Mena *et al* 2023). This is problematic, as approximately 5% of individuals experience hand and wrist pain (Walker-bone *et al* 2004), resulting in long occupational absences (Barr *et al* 2004) and high socioeconomic cost (de Putter *et al* 2012). While FEM has informed treatments in other joints (Favre *et al* 2021, Heller 2022), the paucity of hand and wrist models limits translational benefit.

For instance, scapholunate (SL) interosseous ligament (SLIL) injuries, which are among the most common wrist injuries (Ward and Fowler 2015, Wang *et al* 2022), could be explored using FEM to inform carpal mechanics, progression to SL advanced collapse (SLAC)-pattern osteoarthritis, and clinical decision-making (Watson and Ballet 1984). One study by Leonardo-Diaz *et al*. investigated SLIL injuries in the context of lunate morphology, concluding that the type II lunate-hamate facet conferred greater SL joint stability following SLIL injuries (Leonardo-diaz *et al* 2020). Another study by Kim *et al*. compared FEM against experimentally-obtained joint pressure, finding that SLIL injuries created measurable, though not significant, changes in joint contact mechanics that may contribute to osteoarthritis (Kim *et al* 2024). Still, FEM generalizability may be limited without incorporating individual variation (Andreassen *et al* 2023, Weiss and Gardiner 2001).

Both the Leonardo Diaz *et al*. study, and the Kim *et al*. study, and most wrist FEM manuscripts, used one or two wrist models based on personalized geometries, but with literature-derived material properties without calibration or variation (Guo *et al* 2009, Gislason *et al* 2010, Bajuri *et al* 2013, Varga *et al* 2013, Alonso Rasgado *et al* 2017, Leonardo-diaz *et al* 2020, Marques *et al* 2021, Kim *et al* 2024, Mena *et al* 2025). A lack of personalized material properties, particularly ligament properties, has been shown to significantly increase errors in predicting personalized output metrics (Alonso Rasgado *et al* 2017, Kim *et al* 2024, Mena *et al* 2025) including joint kinematics and dynamics (Andreassen *et al* 2023), but this has not been widely addressed. Moreover, recent recommendations by the American Society of Mechanical Engineers and the Food and Drug Administration heavily focus on uncertainty quantifications for modeling-based studies in medical device evaluation and *in silico* trials (ASME 2018, FDA 2023). Creating models that can efficiently vary parameters is crucial for accurate recreation of joint motion and to instill confidence in models from a regulatory perspective.

Beyond the necessity of these variations for regulatory use, material variations can provide additional benefits. Calibrating material property parameters to experimental data has improved predictive capabilities (Erdemir *et al* 2019, Hume *et al* 2020, Andreassen *et al* 2023, Anantha Krishnan *et al* 2024). Moreover, statistical methods, including Monte Carlo (MC) analysis give insights into the relationships between parameters and model predictions (Baldwin *et al* 2009). However, these hinge on workflows that rapidly develop and execute individualized models. Furthermore, it has been shown that the largest barrier to adopting modeling in clinical settings is the time required (Lesage *et al* 2023). Most workflows require expertise and substantial computational resources, rendering multiple models impractical. While model run-times are not commonly published, single simulations exceeding 242 h have been reported (Gislason *et al* 2010). Considering the small number of samples in most studies, the lack of studies involving material property variation, and the large computation times reported, it is likely that most existing models for the hand and wrist, using existing workflows, are not conducive to rapid development and execution. As such, while rapid performance is commonplace in models of other joints, this feature is limited in hand and wrist models. Addressing the time-intensive nature of FEM is critical.

Our overall goal in this work was to develop a workflow to rapidly create personalized wrist models with short computation times. This workflow enables personalization and variation of model material properties. This study introduces a novel workflow that combines morphing techniques with algorithmic methods to automate the creation of personalized wrist models that generate solutions rapidly. Furthermore, this work demonstrates how these wrist models facilitate studies requiring material property variation relevant to Digital Twins, *in* *silico* clinical trials, and enhanced clinical understanding of both short- and long-term injury implications. The workflow was used to create three personalized models of healthy individuals utilizing dynamic CT collected in prior studies (Trentadue *et al* 2024a, 2025). Two key analyses are presented to demonstrate the impact of this work. In Analysis 1, models recreated the steps to personalize material properties for the individual participants by optimizing model-predicted kinematics to the participants’ experimentally-obtained 4DCT kinematics. In Analysis 2, Monte Carlo methods simulated the effects of SL ligament injuries, considering physiological ligament material property variation, to illustrate connections between altered kinematics, ligament forces, and radioscaphoid joint contact pressure following injury. This work lays the groundwork for future advancements in personalized wrist modeling.

## 2. Methods

**Figure 2.**
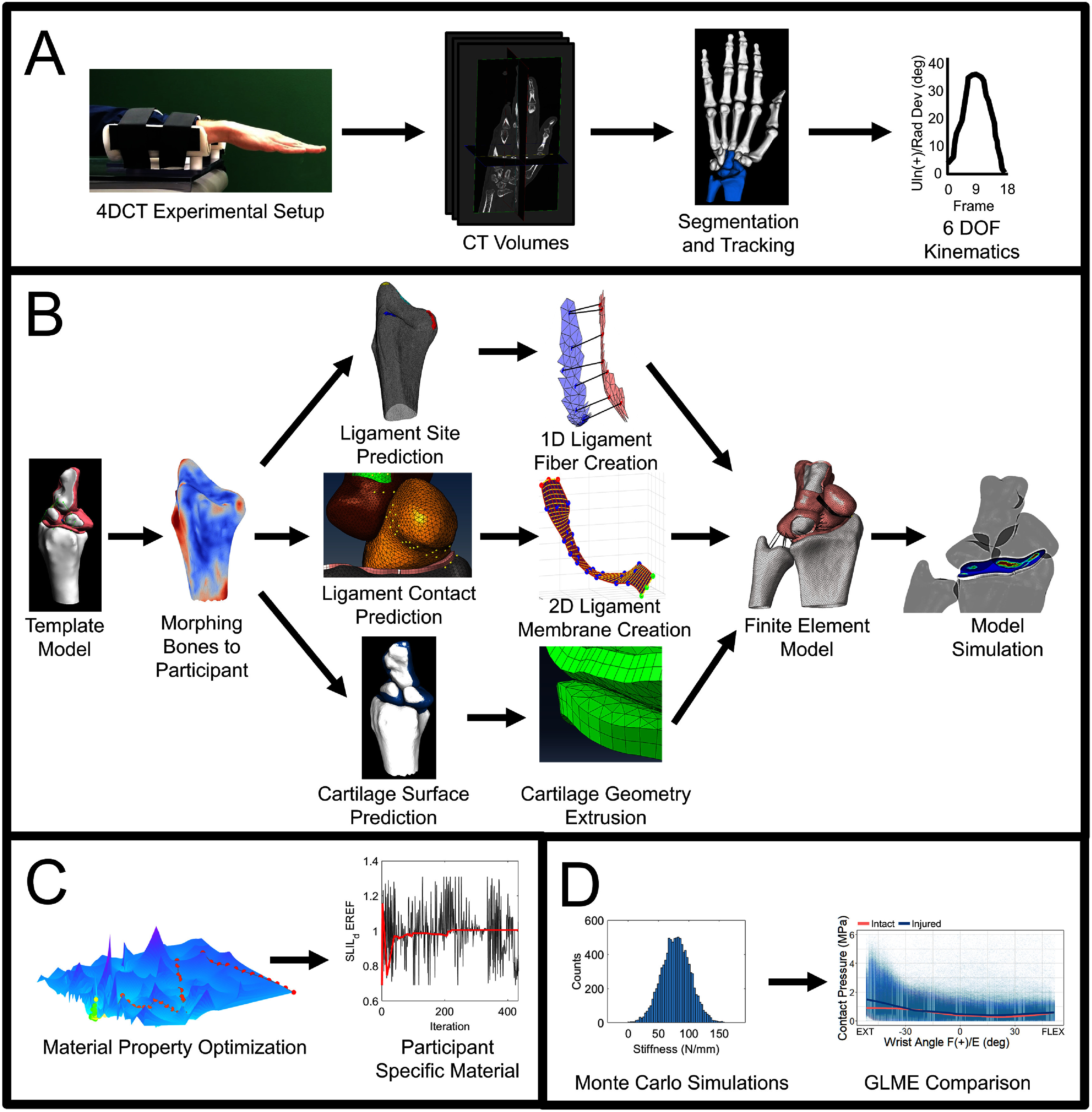
Overall study workflow. (A) Experimental workflow from four-dimensional computed tomography (4DCT) to resulting bone surface geometries and 6DOF joint kinematics. (B) Model development workflow using bone surface geometries and a template model to define personalized ligament and cartilage geometries to create the final finite element model. (C) Ligament material calibration to obtain personalized material properties from minimizing 6DOF kinematic cost (Analysis 1) (D) Monte Carlo (MC) analysis varying ligament material properties and ligament injuries to determine the impacts on joint contact and ligament loads (Analysis 2).

Finite element models were created in Abaqus Explicit (Dassault Systemes, France) with personalized geometries and a range of material properties, boundary conditions, and simulation setups. An overview of the experimental data, details of model development, and analyses performed is outlined (figure 2). Specific Abaqus implementations of elements, materials, boundary conditions, and interactions, as well as a graphical abstract, are included in the Supplementary Materials.

### 2.1. Experimental data

Static and 4DCT wrist images were acquired from asymptomatic participants in an IRB-approved study (NCT04736537) (Trentadue *et al* 2024a, 2025); a subset of three participants is included (table 1). Static CT volumes spanned the distal radial metadiaphysis through the distal phalanges. Participants were randomized to one of three dynamic tasks: flexion-extension (FE), radial-ulnar deviation (RU), and grip. During data collections, bilateral wrists were imaged while performing functional tasks using previously published methods (Trentadue *et al* 2024b). Participants performed tasks first without resistance (unresisted) and then with moderate resistance (resisted). 4DCT captured 15 timepoints per motion cycle with 66 ms temporal resolution. A motion cycle was defined as moving from one extremum to the other and back (Trentadue *et al* 2024b).

**Table 1.** Participants recruited to a study of normative wrist anatomy and biomechanics were imaged using three- and four-dimensional computed tomography (4DCT) (Trentadue *et al* 2024a, 2025). Three participants were used to generate personalized finite element models. The dominant, right hands were modeled for all participants. Type I facets do not have an additional articulation between the lunate and hamate, while Type II facets have an articulation between the lunate and the hamate. Calibration 4DCT motion refers to experimental motion by each participant done without an applied force resisting the motion. In contrast, benchmark 4DCT motion refers to experimental motion by each participant that was done in the presence of a small, applied force resisting the motion.

Participant number	Sex	Age (years)	Lunate-hamate facet type	Calibration 4DCT motion	Benchmark 4DCT motion
1	Female	39	Type I	Unresisted radial-ulnar deviation	Resisted radial-ulnar deviation
2	Male	38	Type I	Unresisted grip	Resisted grip
3	Female	29	Type II	Unresisted flexion-extension	Resisted Flexion-extension

**Figure 3.**
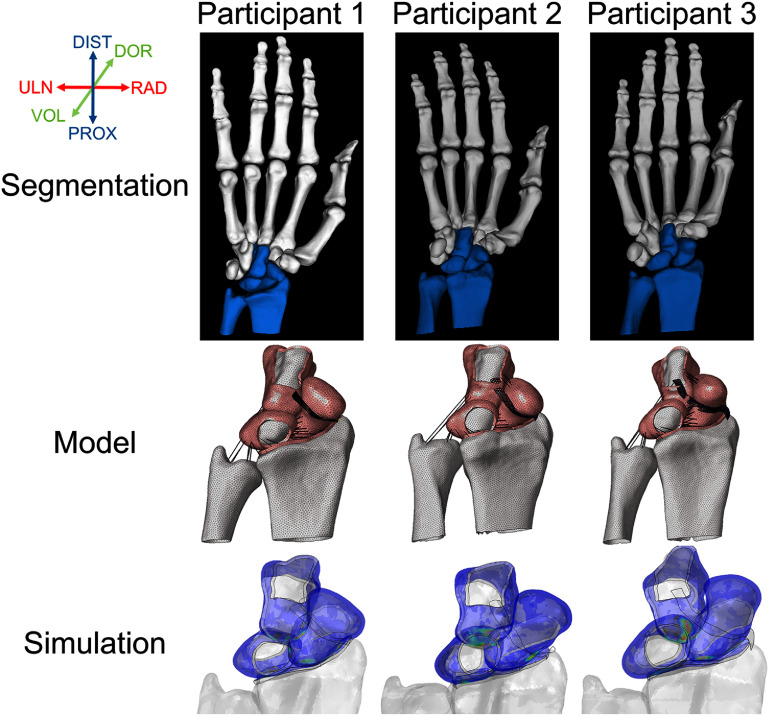
3D bone surfaces generated from segmentation from static CT, finite element model, and model simulation with a sample frame of the contact pressure plot at the neutral pose, representing a radiocapitate extension angle of approximately 0°.

Models were restricted to the radius, scaphoid, lunate, capitate, and ulna. Bones were segmented from static CT in Analyze (Mayo Foundation for Medical Education and Research, Rochester, MN). Segmentations were used to create three-dimensional surfaces (figure 3), exported as stereolithography mesh files. Coordinate systems were assigned to the radius (RCS) using a modified ISB standard coordinate system (Coburn *et al* 2007). Coordinate systems for other bones were defined relative to the RCS in the static image. A custom pipeline was used to produce *n* 4 × 4 homogenous transformation matrices in RCS at each of *n* timepoints per motion arc (Zhao *et al* 2015, Trentadue *et al* 2023b). These 3D geometries and transformation matrices were the basis for this work.

### 2.2. Model development

The novelty of this work was the development of a pipeline to create personalized models from bony geometries alone. Model development is divided into four parts: geometries, material properties, loading and boundary conditions, and model input variation and result extraction.

#### 2.2.1. Geometries

The critical process underlying rapid model development is the automated prediction of soft-tissue geometries by leveraging morphing algorithms and algorithmic steps.

##### 2.2.1.1. Bones

Bone meshes were smoothed and remeshed using open-source software (Moerman 2018) as rigid triangular element surfaces with edge lengths of approximately 1.5 mm.

##### 2.2.1.2. Cartilage

**Figure 4.**
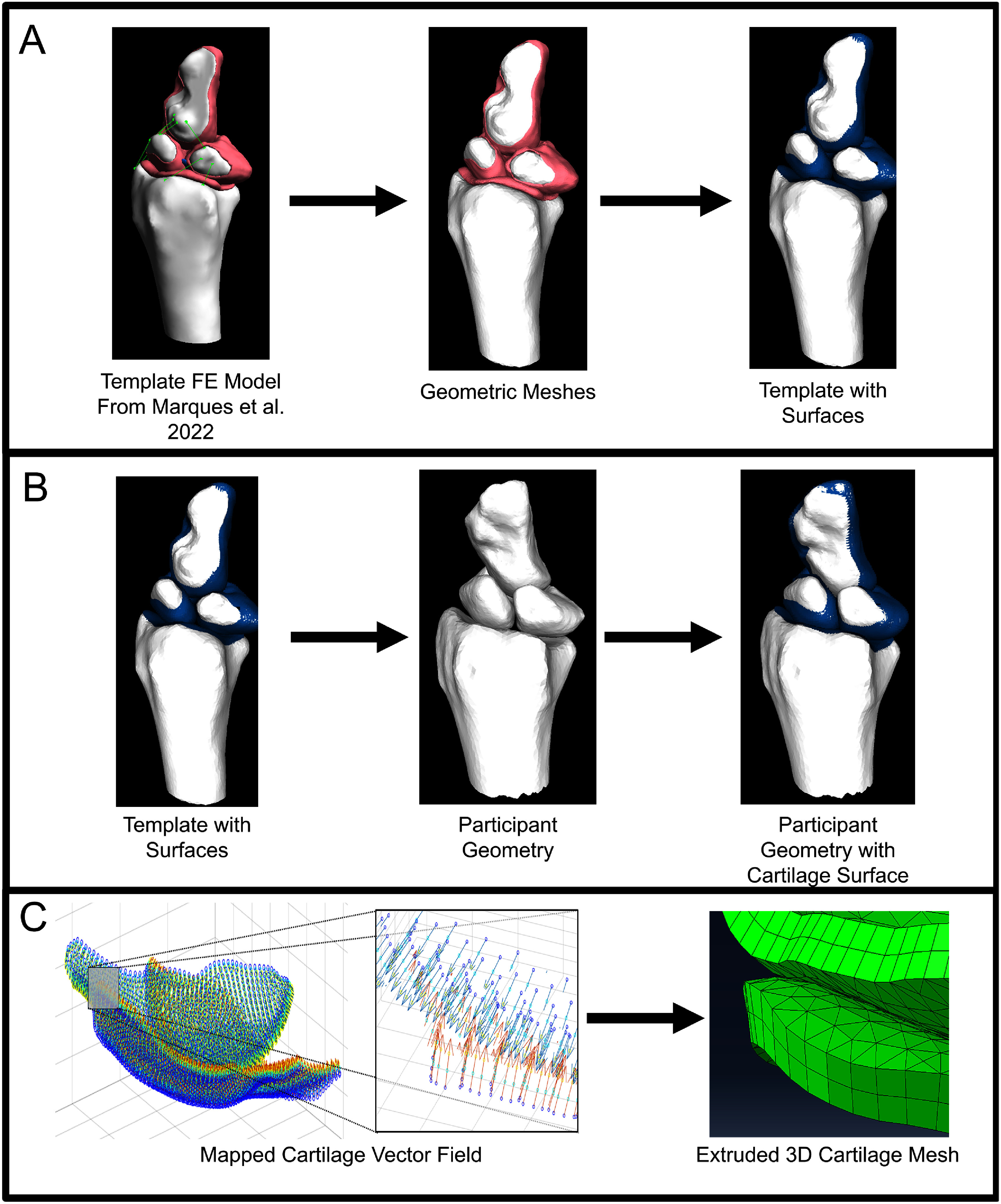
Cartilage 3D mesh creation from approximate surface regions and non-linear mapped extrusion. (A) Creation of cartilage surface (blue) from projection of original 3D mesh to bone surfaces for template geometry. A template bone from Marques *et al*, with previously identified cartilage (pink), was used. 3D cartilage geometries were projected based on their surface normal directions to determine the corresponding cartilage-covered bone surface. (B) Creation of approximate cartilage surface on participant geometry via morphing of template to participant bones and then approximating cartilage region (blue). Template bones with identified cartilage regions were used. These were morphed to each participant’s geometry using validated non-linear morphing algorithms; the solution was used to predict the location of the cartilage surfaces on the new participant’s bones, based on mapped position from the original template mode. (C) Cartilage thickness extrusion to 3D wedge elements via mapped nearest-neighbor distance vector field. The resulting cartilage surfaces on each pair of bones were used to create a distance field to the nearest cartilaginous surface based on all other bones. The distance was used to create 3D mapped neural network solutions for the cartilage thickness and extrude nodal vertices to create 3D wedge elements.

Cartilage was predicted via a two-stage process using MATLAB (R2024a, MathWorks, Natick, MA). In stage 1, cartilage geometries were estimated by morphing a template geometry with known cartilaginous regions from a published model (Marques *et al* 2021) to each participant. In the published dataset, 3D cartilage geometries were projected onto corresponding bones to define cartilage coverage (figure 4(A)). Using a previously validated open-source software package, a morphing algorithm based on generalized regression neural networks (GRNNs), was applied between the target participant and source template geometries (Andreassen *et al* 2024). This algorithm morphs the source template bones to the bones of the target participant, and determines a non-linear mapping between the features on the template bone and target bone respectively. These mappings are then applied to the original cartilaginous regions to predict the location of these regions on the bones of the new participants. The solution was applied to the projected template cartilage geometry and used to predict coverage (figure 4(B)).

In stage 2, these surfaces were used to develop full 3D cartilage elements. Following similar work previously performed in the wrist, a vector field was created from nearest-neighbor distances between surfaces calculated using surface projections (figure 4(C)) (Marai *et al* 2006). A second GRNN was used to define a non-linear mapping between locations on the boney surface and the approximate cartilage width, which was used to extrude nodes (figure 4(C)). These were combined to create 3D triangular wedge elements representing full-thickness cartilage (Gislason *et al* 2010) with edge lengths of approximately 0.35 mm. In some instances, surfaces contained overclosures, which were removed using a combination of HyperMesh (Altair Engineering Inc., Troy, MI) and another overclosure algorithm (Andreassen *et al* 2024) from the same FEMORS package as the morphing algorithm. Mesh quality verification and a mesh convergence study were conducted per recommendations (Anderson *et al* 2007) using FEBio (Maas *et al* 2012) and Abaqus; results are in supplemental material.

##### 2.2.1.3. Ligaments

**Figure 5.**
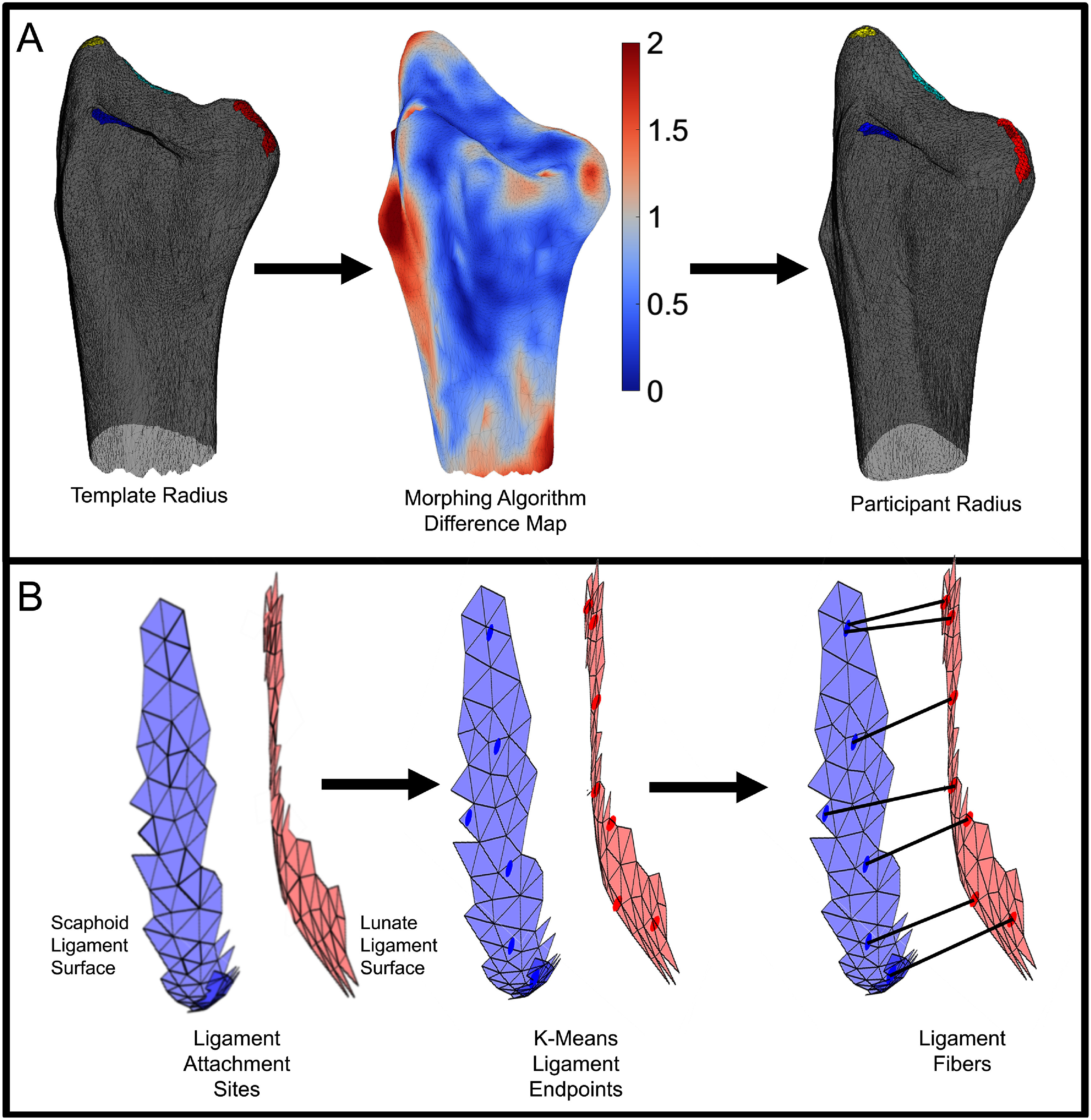
Ligament 1D fiber creation from non-linear morphing and fiber creation. (A) Identification of ligament attachment sites from a template bone followed by non-linear morphing to a participant’s bone (Andreassen *et al* 2024). The difference map shows regions of similarity (blue) and dissimilarity (red) between the template and participant bone. Colored regions represent predicted ligament attachment sites. (B) Creation of ligament fibers from ligament attachment sites on two paired bones. Endpoints are found on each surface using K-means methods. Fibers are created using the Kuhn–Munkres method.

Ligaments were created in a two-stage process (figure 5). In stage 1, a template model with ligament attachment sites was created. Attachment sites were identified using previously modeled ligaments (Marques *et al* 2021) as well as manual identification based on anatomical descriptions (Mizuseki and Ikuta 1989, Berger 2001, Nagao *et al* 2005, Apergis 2013, Moritomo 2013, Ringler 2013, Zhang *et al* 2024) (table 2). This template was morphed to each participant’s bones, using the same processes described for cartilage (Andreassen *et al* 2024), to predict ligament attachment sites on the new participant’s bones (figure 5(A)). Non-linear morphing algorithms have previously been shown to have low errors in predicting physical landmarks and ligament attachments with minimal user involvement (Pillet *et al* 2016, Malbouby *et al* 2025).

**Table 2.** Eleven ligaments were included in the model. Information about the ligament design and material properties is detailed. Parameter values are reported as mean ± standard deviation. The ligament abbreviations are the names of the ligaments reported in the manuscript, while the model abbreviations are the names of the ligaments as defined in the model itself on the relevant repositories.

Ligament name	Ligament abbreviation	Model abbreviation	1D or 2D	Number of fibers	Stiffness (N mm^−1^)	Reference Strain	Allowed failure during MC	Material properties sources	Ligament descriptions sources
Volar scapholunate interosseous ligament	VSL	SLIL_v	1D	7	33 ± 10	1.0 ± 0.1	Yes	Nikolopoulos *et al* (2011)	Berger *et al* (2001)
Proximal (membranous) scapholunate interosseous ligament	PSL	SLIL_p	1D	7	25 ± 7.5	1.0 ± 0.1	Yes	Nikolopoulos *et al* (2011)	Berger *et al* (2001)
Dorsal scapholunate interosseous ligament	DSL	SLIL_d	1D	7	79 ± 24	1.0 ± 0.1	Yes	MetaAnalysis of values in Chang *et al* (2022)	Berger *et al* (2001)
Radioscaphocapitate ligament	RSC	RSC	2D	N/A	10 ± 3	1.0 ± 0.1	Yes		Berger *et al* (2001)
Long radiolunate ligament	LRL	LRL	1D	4	75 ± 23	1.0 ± 0.1	Yes	Bajuri *et al* (2013)	Berger *et al* (Berger 2001), Nagao *et al* (2005), Zhang *et al* (2024)
Short radiolunate ligament	SRL	SRL	1D	4	75 ± 23	1.0 ± 0.1	No	Bajuri *et al* (2013)	Nagao *et al* (2005)
Dorsal radiocarpal ligament	DRC	DRC	1D	3	75 ± 23	1.0 ± 0.1	No		Nagao *et al* (2005), Mizuseki *et al* (1989)
Radial collateral ligament	RCL	RCL	1D	3	10 ± 3	1.0 ± 0.1	No	Bajuri *et al* (2013)	Ringler *et al* (Ringler 2013)
Scaphocapitate ligament	SCL	SCL	1D	7	40 ± 12	1.0 ± 0.1	No	Bajuri *et al* (2013)	Apergis *et al* (Apergis 2013)
Ulnocapitate ligament	UCL	UCL	1D	3	75 ± 23	1.0 ± 0.1	No		Moritomo *et al* (2013)
Ulnolunate ligament	UL	UL	1D	3	40 ± 12	1.0 ± 0.1	No	Bajuri *et al* (2013)	Nagao *et al* (2005)

In stage 2 individual ligament fibers are created using algorithmic techniques. The number of ligamentous fibers was manually defined (table 2) to create similar fiber density, i.e. number of fibers per area, across ligaments. Individual fiber endpoints were determined using a K-means algorithm. Fibers were automatically created using the Kuhn–Munkres algorithm to connect ligament endpoints to create fibers with approximately uniform length fibers (figure 5(B)). The average fiber length was recorded for use in the material model. Ligaments were modeled as 1D non-linear connector elements.

The radioscaphocapitate (RSC), which wraps around the distal scaphoid and has non-uniform properties along its length (Berger 2001), was modeled as a 2D fiber-reinforced membrane (Baldwin *et al* 2012). Ligament attachments on the radius and capitate, and the scaphoid contact region were identified. Curves connecting these regions were used to create a quadrilateral mesh surface with the desired element size. The final mesh had quadrilateral membrane elements with 1D non-linear connector elements connected between quadrilateral elements; a figure showing this is provided in the supplemental material.

#### 2.2.2. Material properties

Rapid model simulation is facilitated by material properties that convert time-consuming material non-linearities into tuned tabular approximations. These approximations exhibit similar behavior but with improved computational speed.

##### 2.2.2.1. Bones

As the focus of this work is recreating bone motion, rather than stress distributions, bone deformation is negligible. In line with prior work for other joints (Rooks *et al* 2021), bones were modeled as rigid bodies with their motion restricted to 6 degrees of freedom (DOF) of a single rigid body node (RBN).

##### 2.2.2.2. Cartilage

Following prior work in the knee and hip (Halloran *et al* 2005, Huff *et al* 2020) cartilage was modeled as a rigid material. However, a contact pressure-overclosure relationship defined between surfaces was defined using a piecewise tri-linear relationship defined between surfaces (Andreassen *et al* 2025a) to enable non-linear contact interactions between cartilage articular surfaces. The coefficients for this piecewise tri-linear contact pressure-overclosure relationship were calibrated following steps from prior work (Halloran *et al* 2005, Andreassen *et al* 2025a) with details elaborated in the supplemental material; however, a summary is provided herein.

The cartilage was modeled as a non-linear deformable hyperelastic material defined using literature-reported values (Mooney–Rivlin material, *C*_10_ = 4.1, *C*_01_ = 0.41, *D*_1_= 0.0475) and evaluated by simulating a controlled compression of the cartilage meshes into one another (Li *et al* 2007, Gislason *et al* 2010) and obtaining the force–displacement curve and overall contact area as a function of load. A near identical model was created with cartilage modeled as rigid with a tri-linear pressure-overclosure relationship applied. The coefficients for the piecewise tri-linear contact pressure-overclosure were calibrated by minimizing the model-predicted force–displacement curve against the one obtained from the non-linear deformable model. The contact area predicted by the calibrated cartilage model was verified against that for the non-linear deformable model. Modeling the cartilage as a rigid material with a calibrated contact pressure-overclosure relationship imparts the model with the behaviors and contact prediction accuracy of a non-linear deformable material but with the improved computational speed inherent to rigid material definitions. While this approach does not permit rapid internal material stress calculations, it does permit quantifications of key contact outcomes for area, pressure, and force (Huff *et al* 2020, Andreassen *et al* 2025a) which are the features of primary interest in this work.

##### 2.2.2.3. Ligaments

Ligaments were modeled with a non-linear tension-only force–displacement curve. This approach to ligament modeling has been established in previous musculoskeletal models (Blankevoort *et al* 1991, Yu *et al* 2001, Andreassen *et al* 2023) wherein the behavior has been defined as follows: the resistance force is zero up to a defined threshold displacement, the force increases quadratically (toe-in region) up to the next defined displacement value, at which point resistance increases linearly unbounded. For each displacement in the material model, the curve is continuous, and continuously differentiable. Each ligament is modeled with a literature-derived constant toe-in strain parameter (0.03) (Butler *et al* 1986) and ligament-specific reference length, stiffness, and reference strain. Reference length was defined as the average ligament bundle length. Stiffness and reference strain were design variables, allowing for variation in material properties.

In this work, the stiffness parameter represents the slope of the linear region of the force–displacement curve, analogous to stiffness values commonly reported in cadaveric ligament testing. The reference strain material parameter defines the total change in length required before the ligament generates resistance force. Reference strain values greater than 1.0 indicate that the ligament is initially taut, and therefore already loaded in its initial model position, where the bones are aligned with the participant’s imaging scan. In contrast, reference strain values less than 1.0 indicate that the ligament is initially slack and therefore needs additional stretch before it generates resistance force. While ligament stiffness is frequently included in models of the hand and wrist, inclusion of initial ligament slack or tension, i.e. reference strain, to our knowledge, has not previously been included in such models (Mena *et al* 2023). Where available, literature-derived mean ±3 standard deviation (SD) ligament stiffness ranges were used (Nikolopoulos *et al* 2011, Bajuri *et al* 2013, Chang and Suh 2022). Unavailable SDs were estimated using a coefficient of variation of 0.3. As reference strain ranges in the wrist are underreported, they were estimated based on work in the knee. As reference strain across all knee ligaments was 0.96 ± 0.13, rarely exceeding 1.30 (Zhang *et al* 2021, Andreassen *et al* 2023), the parameter ranges were set at 1.00 ± 0.30. Reference strain values exceeding 1.00 represent tension in the base pose of the model, while values under 1.00 represent slack.

All ligament fibers within a bundle were given equivalent material properties. Ligaments were modeled with a parameter equation to allow for simulated failure (Andreassen *et al* 2025a). This equation defines any negative scalar in the parameter equation to result in immediate failure of all fibers of a given ligament and removing them from the model thereby simulating injury. In this work, ligament injury represents a complete tear of the ligament. For the dorsal SLIL, complete tears are prevalent in 78% of cases (Andersson and Garcia-elias 2013).

#### 2.2.3. Loading and boundary conditions

The radius and ulna were completely fixed, while the lunate and scaphoid were free in all DOF. Capitate motion differed between the two types of analyses performed. In Analysis 1, capitate motion used experimentally obtained kinematics, while Analysis 2 used idealized joint kinematics. Cartilage and ligaments were rigidly tied to the bone RBNs. A set of nodes aligned to the local coordinate system for each bone was also tied to the RBNs and used to calculate bone kinematics during post-processing.

Initial bone positions were defined from static CT and then models performed two steps: settling and motion. During the settling step, injured ligaments were given a failure condition and removed. Initial resting ligament tension, a result of the applied reference strain, was applied to settle positions of the lunate and scaphoid. The capitate was moved from its static CT pose to its appropriate starting position. In the second step, capitate motion was applied; bone kinematics and results were extracted.

##### 2.2.3.1. Experimentally obtained kinematics

In the first type of joint simulation, models recreated experimentally obtained participant capitate motion, relative to the radius, for each participant from their respective 4DCT data. The 4 × 4 transformation matrices of the capitate relative to the radius from the experimental motion were converted to 6DOF kinematics of the capitate relative to the radius which included the 3 cartesian translations of the RBN as well as 3 rotations calculated as Rodrigues angles. Each value was then offset by the 6 DOF values for the capitate relative to the radius in the initial configuration of the model (static CT pose) to obtain the net change in translation and rotation of the capitate during a given motion. These time-dependent trajectories were then applied in the Abaqus models using a displacement boundary condition applied to the RBN of the capitate for each DOF.

##### 2.2.3.2. Idealized simulated kinematics

In the second type of joint simulation, models recreate an idealized joint motion, rather than matching an exact motion obtained from an individual participant. Models applied idealized wrist flexion-extension kinematics to the capitate. A smooth ramped trajectory was created from maximum flexion (50 deg.) to maximum extension (−50 deg.). This curve was then offset by the initial value for the flexion-extension for the models in the initial configuration of the model (static CT pose). This time-dependent flexion-extension trajectory was then applied in the Abaqus models using a displacement boundary condition applied to the RBN of the capitate in the flexion-extension boundary condition direction. All other rotational DOF were kept constant at their initial rotation and similarly applied using a displacement boundary condition to the capitate RBN. To allow the capitate to maintain contact with the proximal row, all translational DOF were left in zero-load control except for the compressive axis, which applied a small load to ensure contact.

#### 2.2.4. Model input variation and results extraction

##### 2.2.4.1. Run time material variation

27 Ligament design variables—22 ligament material properties and 5 parametric scalars controlling the parametric failure equations for the subset of ligaments designed to simulate injury (table 2)—were defined for each analysis. The ligaments selected to include the model of failure coincide with those that are frequently associated with the progression of SLIL injury severity (Trentadue *et al* 2023a, Zhou *et al* 2024). Design variables were updated using a previously developed custom automated software interface (Hume *et al* 2020, Andreassen *et al* 2023).

##### 2.2.4.2. Model output extraction

FEM results were extracted using custom code inspired by Hume *et al* (2020). Briefly, a custom set of MATLAB and Python scripts are used to extract model results using the Abaqus API. The Python script extracted the Cartesian locations of the nodes tied to each bone defining its local coordinate system axes, as well as the general contact metrics and ligament forces at all timepoints and exported these to a temporary file. The MATLAB script then loads this temporary file and converts the nodal locations into corresponding 4 × 4 homogenous transformation matrices for each bone and organizes all the relevant output metrics of interest. All captured outputs were combined with the original 27 ligament material design variables for the iteration and appended to a log file of all simulation iterations. This allows future analyses to investigate all captured values.

### 2.3. Analyses

Two different analyses demonstrate how models can be applied to clinically relevant questions. In Analysis 1, ligament material properties were calibrated to match model predictions of joint motion to experimental data. In Analysis 2, ligament material properties were varied using MC analysis to evaluate effects of ligament injury on joint contact pressure while controlling for variation in material properties. All analyses were performed on a standard computer using four parallel cores on the central processing unit (Intel Core i5-12 600 Processor). The analysis relied on standard hardware for broader applicability. While the exact memory usage varied greatly and was based on several factors, it averaged around 300 MB for each model simulation and never exceeded 500 MB.

#### 2.3.1. Analysis 1—personalized ligament material calibration

This analysis demonstrates a method to calibrate ligament material properties of each model to best match the experimentally obtained motion of individuals towards development of patient-specific models. This allows for quantification of individualized loads, joint contact, etc. The analysis includes comparisons of calibrated material properties and model performance of kinematic predictions between the models with calibrated material properties and those with average material properties.

##### 2.3.1.1. Calibration steps

Capitate motion was driven with experimentally obtained 4DCT kinematics during the unresisted joint motion, while motion of the scaphoid and lunate were calibrated to match their measured motion. Based on previous methods, an error transformation matrix was calculated between the model-observed and the experimentally obtained motion for the scaphoid and lunate (Hamilton *et al* 2022). The magnitude of error was calculated for each DOF. The 75th percentile of error across all frames was used as a single scalar estimate of error for each DOF (Andreassen *et al* 2025a). Errors were normalized by the range of experimental kinematic values for each DOF. Normalized errors were scaled by corresponding weights based on the relevance of each DOF. The resulting weighted errors were summed across both the lunate and scaphoid to estimate overall cost. Calibration algorithms then iteratively adjust ligament material properties to minimize this cost. Calibration was performed first using surrogate optimization and then, following convergence, a local calibration based on gradient descent.

##### 2.3.1.2. Simulations

Calibration of material properties was performed for all models to their respective unresisted motion (table 1). Calibration iterations logged scaphoid and lunate kinematics relative to the radius in all 6 DOF at each time step, as well as contact metrics and ligament forces for all ligaments. The calibrated material properties (reference strain and stiffness) were also recorded.

##### 2.3.1.3. Comparisons

The calibrated material properties for each participant were compared to identify inter-participant differences. Values were also normalized to their respective *Z*-score ranges from the mean and SD for each value. Final calibrated models were compared against experimentally obtained kinematics for the scaphoid and lunate for all participants. Firstly, to test the overall accuracy of the models, model predicted kinematics and experimental data during the calibrated unresisted motion (table 1) were compared, for both the final models (calibrated material properties) and the original models (material properties from population averages). Additionally, the robustness of the models was evaluated with a benchmarked activity (table 2) using the participant’s resisted motion. These motions were deliberately not used for calibration and thereby test the model’s ability to make predictions beyond what it is explicitly calibrated to. Again, comparisons were made for both the final and the original models. Root mean squared errors (RMSE) between model and experimental measures were calculated. This comparison is analogous to the training (calibration) and validation or testing (benchmarking) frequently performed in other fields, such as machine learning.

#### 2.3.2. Analysis 2—ligament MC analysis

This analysis demonstrates a way to use MC analysis to investigate the effects of ligament injuries while accounting for variations in ligament material properties. Simulating injuries to ligaments can inform the sensitivity of radioscaphoid contact pressure to these injuries. Additionally, MC analysis can investigate ligament forces, something that is impractical from experimental methods alone.

##### 2.3.2.1. MC steps

Joint motions were predicted with unique combinations of material variation and ligament injury. The models applied idealized FE motion to the capitate during each simulation with material properties randomly chosen. For ligaments commonly associated with SL injuries (table 2), the scalars for injury were randomly sampled, with a 30% chance of failure. Each ligament injury was simulated independently, allowing for all combinations to be simulated. All random values (material properties and failure conditions) were chosen using Latin hypercube sampling. Simulations recorded the combination of injured ligaments and radioscaphoid contact pressure. Upon visual inspection, models with contact pressures exceeding 16.5 MPa yielded unrealistic simulations; 68 simulations (0.27%) were therefore excluded, but simulations were repeated until 12 500 successful simulations were performed.

##### 2.3.2.2. Simulations

Models of the age- and sex-matched participants (Participant 1 and 2) were included in the MC analysis with a total of 25 000 simulations (12 500 each). Each simulation included values for all capitate FE angles. As such, approximately 5 million datapoints (201 FE angles for 25 000 simulations) were obtained for analyses.

##### 2.3.2.3. Comparisons

The median rotational kinematics of the scaphoid relative to the radius were quantified and compared between participants and between simulations with all ligaments intact and those with at least one ligament injury. In addition, the total variation of the datapoints at each capitate flexion-extension angle was quantified as the average difference between the 1st and 99th percentiles of the rotational kinematics across all simulations for the entire capitate flexion-extension range of motion. Radioscaphoid contact pressures were analyzed across all simulations using generalized linear mixed effects (GLME) models in R (R Foundation for Statistical Computing, Austria). GLME models predicted radioscaphoid contact pressure as a function of the wrist flexion-extension angle and the presence of ligament injuries. flexion-extension angle and ligament injury states were fixed effects. Combinations of material properties were treated as random effects. Significance was calculated using *t*-tests as the standard approach for GLME models. Given the large total sample size, but a limited number of unique subjects (*n* = 2), the minimum level of statistical significance, *α*, was set to *α* = 0.001 to minimize the chance of false positives. Radioscaphoid contact pressures were compared with previous wrist models (Kim *et al* 2024, Mena *et al* 2025). For intact simulations, ligament forces in the volar, proximal, and dorsal SLIL were quantified, and GLME models were used to determine differences.

## 3. Results

### 3.1. Computational speed

#### 3.1.1. Model development

Following workflow development, the time required to create a new personalized model, excluding bone segmentation, was under two hours of unsupervised computation.

#### 3.1.2. Model simulation

Each MC iteration took approximately 45 s, totaling approximately 13 d of unsupervised computation time for all 25 000 simulations (Analysis 2). Material calibration iterations required more time to converge with experimental kinematics, approximately 145 s (about 2.5 min) for 275 iterations. The total calibration time was approximately 11 h for each model (Analysis 1).

### 3.2. Analysis 1—personalized ligament material calibration

#### 3.2.1. Material properties

**Figure 6.**
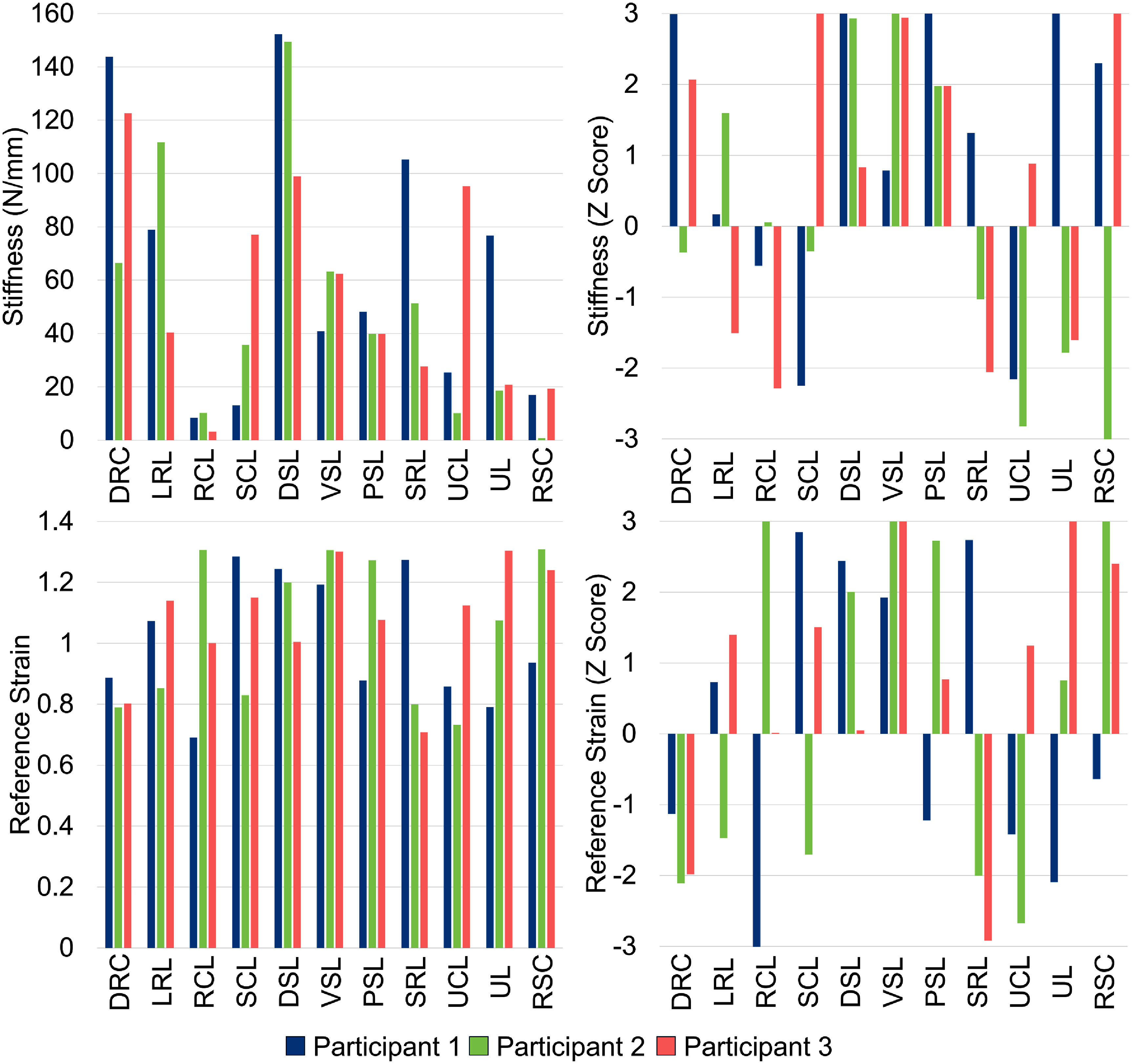
Model-derived calibrated ligament material properties. Reference strain values greater than 1.0 represent initial tension in the static CT-derived neutral wrist position. Values reported as true metrics (left) and *Z*-score normalized to the mean and standard deviation for each material property (right). The ligament properties determined were calculated for all modeled ligaments which include the following: dorsal radiocarpal ligament (DRC), long radiolunate ligament (LRL), radial collateral ligament (RCL), scaphocapitate ligament (SCL), dorsal scapholunate interosseous ligament (DSL), volar scapholunate interosseous ligament (VSL), proximal (membranous) scapholunate interosseous ligament (PSL), short radiolunate ligament (SRL), ulnocapitate ligament (UCL), ulnolunate ligament (UL), radioscaphocapitate ligament (RSC). Note, material properties in the UCL did not converge to a single final value as this ligament connected two bones that were completely restricted in displacement control, namely the ulna and capitate. As such, material properties for this ligament had no impact on the optimization and are therefore likely inaccurate.

Ligament stiffness and reference strain values were unique to individuals and varied greatly (figure 6). Patterns within and across ligaments were more difficult to observe based on raw values alone, but patterns were more easily identified using the *Z-*Scores for the stiffnesses and reference strains calculated relative to the literature reported values in table 2.

##### 3.2.1.1. Ligament stiffnesses

Stiffnesses across ligaments varied between 2 N mm^−1^ at the lowest (RSC in Participant 2) to 153 N mm^−1^ at the greatest (DSL in Participant 1). When normalized to *Z*-Scores, the stiffnesses of the SLIL bundles (DSL, VSL, and PSL) were all greater than the average stiffness of these ligaments reported in the literature (table 2). Additionally, some ligaments like the SRL and LRL varied in terms of stiffness, but when normalized to *Z*-score varied much less. In contrast, ligaments like the RSC varied little in terms of stiffness, but, when normalized to *Z*-score, it reached opposite extremes of ±3 for Participants 2 and 3.

##### 3.2.1.2. Ligament reference strains

Reference strains were between 0.69 at the smallest to 1.30 at the greatest, but both extremes were found in the RCL (figure 6). However, the maximum reference strain of 1.30 was also found for the VSL and RSC for Participant 2. When normalized to *Z*-scores, the VSL had reference strains greater than 1.0, while the DRC ligament had reference strains less than 1.0 for all participants. In addition, the RCL ligament was found to have achieved opposite extremes of ±3 for Participants 1 and 2.

#### 3.2.2. Personalized model kinematic errors

##### 3.2.2.1. Calibrated motion

**Figure 7.**
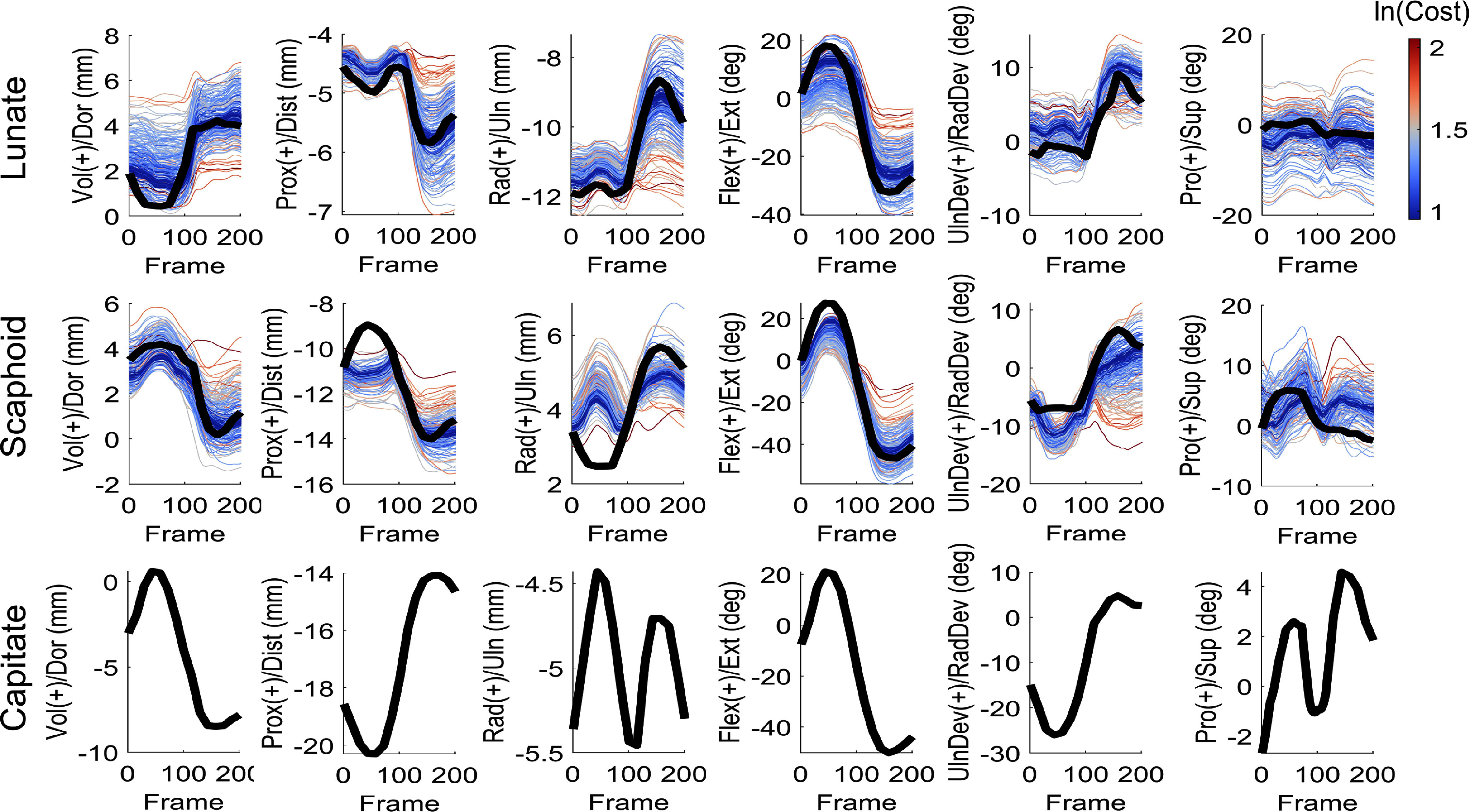
Kinematic predictions from Participant 3 during model calibration of participant unresisted flexion-extension motion. Kinematics were driven for the capitate in displacement control, while the lunate and scaphoid were free to move. Solid black lines represent experimentally observed 4DCT motion. All other lines represent a single calibration iteration, with lines in red having the greatest cost, and lines in blue having the smallest cost. Costs included are a weighted sum of the accuracy of scaphoid and lunate translational and rotational kinematics relative to experiment. Final calibrated ligament parameters for model calibration are from the simulation with the lowest total cost (darkest blue line). The degrees of freedom modeled are the following: volar-dorsal (vol/dor) translation, proximal-distal (prox/dist) translation, radial-ulnar shift (rad/uln) translation, flexion-extension (flex/ext) rotation, ulnar-radial deviation (ulndev/raddev) rotation, and pronation-supination (pro/sup) rotation. Positive sign convention is tagged (+) in the *y*-axis label for the relevant direction. For example, the label ‘Vol(+)/Dor (mm)’ indicates that the sign convention defines volar direction motion to be positive, while the dorsal direction motion is negative.

Calibration of ligament parameters resulted in better recreation of non-linear motion trajectories for both the scaphoid and the lunate (figure 7). As calibration decreased the cost, the fit of the lines qualitatively improved, and resulted in a decreased RMSE over the original model with population average material properties. In the flexion-extension rotation for Participant 3, calibration decreased the RMSE of the scaphoid from 9.55 deg. to 5.75 deg. (figure 7), and for the lunate from 10.93 deg. to 5.14 deg. In the proximal-distal translation, calibration decreased the RMSE from 1.66 mm to 0.93 mm for the scaphoid and from 0.21 mm to 0.10 mm for the lunate.

In general, calibration decreased the overall RMSE between model predicted motion and experimentally observed motion compared to the population models (table 3), but the degree of improvement varied across DOF and differed between bones. Lower RMSEs for the scaphoid were observed compared to the lunate and for translations compared to rotations. For translations, maximum RMSE was 1.7 mm and 1.3 mm for the population and calibrated models, respectively. The median [5th, 95th percentile] change in translational RMSE for the calibrated trial relative to the population trial was −0.1 [−0.3, 0.4] and −0.2 [−0.7, 0.5] mm for the scaphoid and lunate, respectively. For rotations, maximum RMSE was 10.9 deg. and 5.75 deg. for the population and calibrated models, respectively. The median [5th, 95th percentile] change in rotational RMSE for the calibrated trial relative to the population trial was −1.2 [−4.0, 1.3] deg. and −0.4 [−5.8, 1.7] deg. for the scaphoid and lunate, respectively.

**Table 3.** Root mean squared error (RMSE) of personalized model kinematic predictions with ‘population’ average material properties and calibrated material properties against experimentally obtained kinematics during the calibrated unresisted joint motion. The degrees of freedom modeled are the following: volar-dorsal (vol/dor) translation, proximal-distal(pro/dis) translation, radial-ulnar shift (rad/uln) translation, flexion-extension (flex/ext) rotation, ulnar-radial deviation (ulndev/raddev) rotation, and pronation-supination(pro/sup) rotation. Calibration motion refers to the model’s ability to recreate experimental data in the calibrated motions.

			Translation RMSE (mm)			Rotation RMSE (deg.)		
Participant	Bone	Material	Vol/Dor	Pro/Dis	Rad/Uln	Flex/Ext	UlnDev/RadDev	Pro/Sup
1	Scaphoid	Population	0.31	0.52	0.23	3.84	6.13	2.62
		Calibrated	0.22	0.41	0.27	2.21	2.13	1.45
	Lunate	Population	0.93	0.46	0.69	5.11	3.25	2.96
		Calibrated	1.33	0.55	0.69	5.70	3.68	4.26

2	Scaphoid	Population	0.45	0.43	0.31	4.54	1.03	2.32
		Calibrated	0.13	0.09	0.02	0.99	0.23	0.32
	Lunate	Population	0.86	0.02	0.22	5.21	5.14	2.66
		Calibrated	0.91	0.02	0.05	5.13	5.29	1.39

3	Scaphoid	Population	0.43	1.66	1.21	9.55	5.81	3.76
		Calibrated	0.97	0.93	0.78	5.75	3.09	3.52
	Lunate	Population	1.31	0.21	0.95	10.93	1.19	3.07
		Calibrated	0.65	0.10	0.31	5.14	2.88	2.72

##### 3.2.2.2. Benchmark motion

Benchmarking, in contrast to the calibrated motions, tests the model’s ability to predict unseen joint motions, in this case, the experimental resisted joint motion for each participant (table 1). Generally, improvements in the benchmark motions were found across most DOF, but the improvements were lower in magnitude than those of the calibrated unresisted motion. While the maximum RMSE across all DOF and participants was greater for the calibrated model compared to the population model in the benchmark cases, the majority of DOF improved with calibration across all models (table 4). For translations, maximum RMSE was 1.15 mm and 1.68 mm for the population and calibrated models, respectively. The median [5th, 95th percentile] change in translational RMSE for the calibrated trial relative to the population trial was 0.0 [−0.6, 0.5] and −0.1 [−0.7, 0.8] mm for the scaphoid and lunate, respectively. For rotations, the maximum RMSE was 9.40 deg. and 10.73 deg. for the population and calibrated models, respectively. The median [5th, 95th percentile] change in rotational RMSE for the calibrated trial relative to the population trial was −0.8 [−2.3, 0.9] deg. and −0.1 [−3.3, 2.3] deg. for the scaphoid and lunate, respectively.

**Table 4.** Root mean squared error (RMSE) of personalized model kinematic predictions with ‘population’ average material properties and calibrated material properties against experimentally obtained kinematics during the benchmark resisted joint motion. The degrees of freedom modeled are the following: volar-dorsal (vol/dor) translation, proximal-distal(pro/dis) translation, radial-ulnar shift (rad/uln) translation, flexion-extension (flex/ext) rotation, ulnar-radial deviation (ulndev/raddev) rotation, and pronation-supination(pro/sup) rotation. Benchmark motion refers to the model’s ability to recreate experimental data in the unseen benchmark motions.

			Translation RMSE (mm)			Rotation RMSE (deg.)		
Participant	Bone	Material	Vol/Dor	Pro/Dis	Rad/Uln	Flex/Ext	UlnDev/RadDev	Pro/Sup
1	Scaphoid	Population	0.67	0.67	0.67	4.47	4.68	3.74
		Calibrated	0.49	0.67	0.65	3.68	2.75	3.10
	Lunate	Population	1.14	0.51	0.76	6.49	3.31	3.07
		Calibrated	1.68	0.60	1.08	6.67	4.23	3.38

2	Scaphoid	Population	1.02	1.14	0.67	9.40	3.38	6.22
		Calibrated	0.76	0.56	0.33	7.11	1.57	3.96
	Lunate	Population	1.15	0.02	0.69	8.48	6.57	6.34
		Calibrated	1.47	0.11	0.24	10.73	6.45	5.74

3	Scaphoid	Population	0.23	1.29	0.91	4.78	7.13	2.39
		Calibrated	1.03	0.83	0.53	3.40	3.80	2.51
	Lunate	Population	0.58	0.11	1.15	8.30	2.09	2.97
		Calibrated	0.44	0.08	0.44	5.60	2.26	3.64

#### 3.2.3. Ligament forces

**Figure 8.**
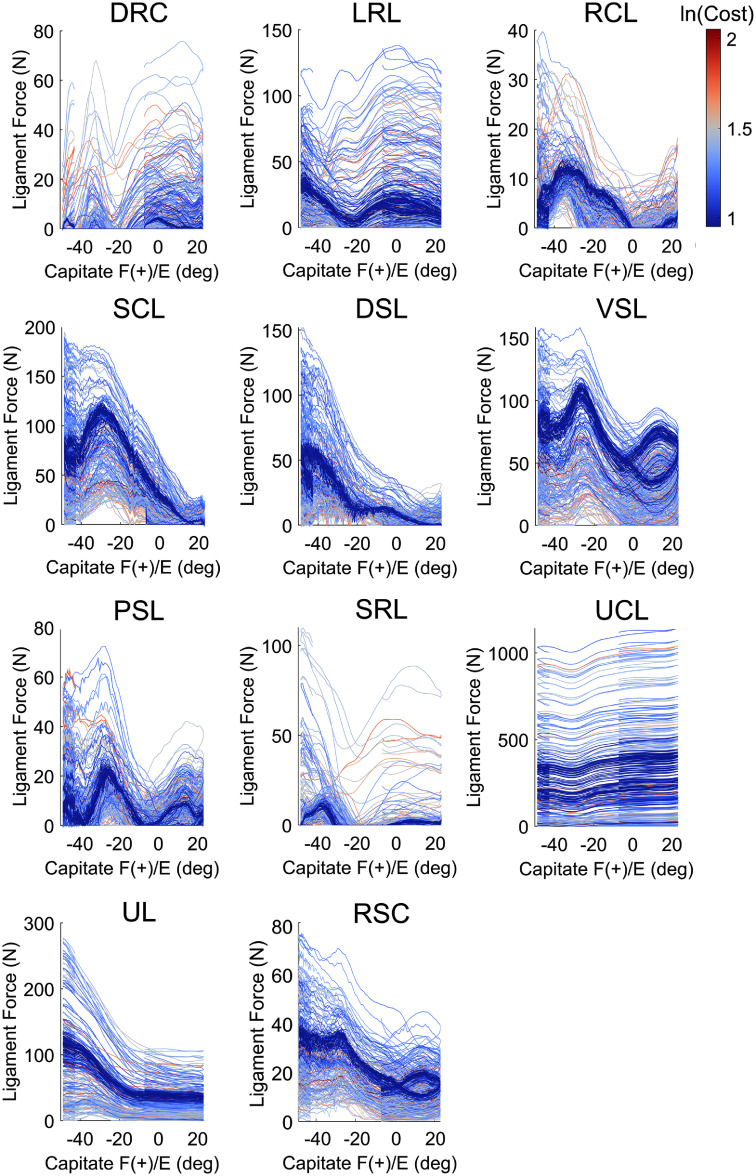
Predictions of ligament forces from participant 3 during model calibration of participant unresisted flexion-extension motion. Ligament forces are plotted against the capitate flexion-extension rotation angle during the full motion cycle. All lines represent a single calibration iteration, with lines in red having the greatest cost, and lines in blue having the smallest cost. Final calibrated ligament parameters for model calibration are from the simulation with the lowest total cost. The ligament properties determined were calculated for all modeled ligaments which include the following: dorsal radiocarpal ligament (DRC), long radiolunate ligament (LRL), radial collateral ligament (RCL), scaphocapitate ligament (SCL), dorsal scapholunate interosseous ligament (DSL), volar scapholunate interosseous ligament (VSL), proximal (membranous) scapholunate interosseous ligament (PSL), short radiolunate ligament (SRL), ulnocapitate ligament (UCL), ulnolunate ligament (UL), radioscaphocapitate ligament (RSC). Note, forces in the UCL did not converge to a single final value as this ligament connected two bones that were completely restricted in displacement control, namely the ulna and capitate. As such, material properties for this ligament had no impact on the optimization, and therefore the ligament forces in the UCL are likely inaccurate.

Ligament forces for the Participant 3 model during each iteration are shown in figure 8 for their calibration motion, namely unresisted wrist flexion and extension. As the cost was minimized, the ligament forces stabilized to unique values for each ligament across the range of motion. For most ligaments, the forces increased with increasing capitate extension. Additionally, the final calibrated iteration for the VSL had a force that was nearly double that of the force in the DSL, with the VSL reaching a peak force of 109.1 N while the DSL reached a peak of 58.2 N across the motion. Both the DSL and VSL forces across the range of motion were greater than the force in the PSL which reached a peak of 22.2 N. Forces in the UCL did not converge to a single final value as this ligament connected two bones that were completely restricted in displacement control, namely the ulna and capitate. As such, material properties for this ligament had no impact on the optimization, and therefore the ligament forces in the UCL are likely inaccurate.

### 3.3. Analysis 2—ligament property MC analysis

#### 3.3.1. Scaphoid kinematics

##### 3.3.1.1. Intact

**Figure 9.**
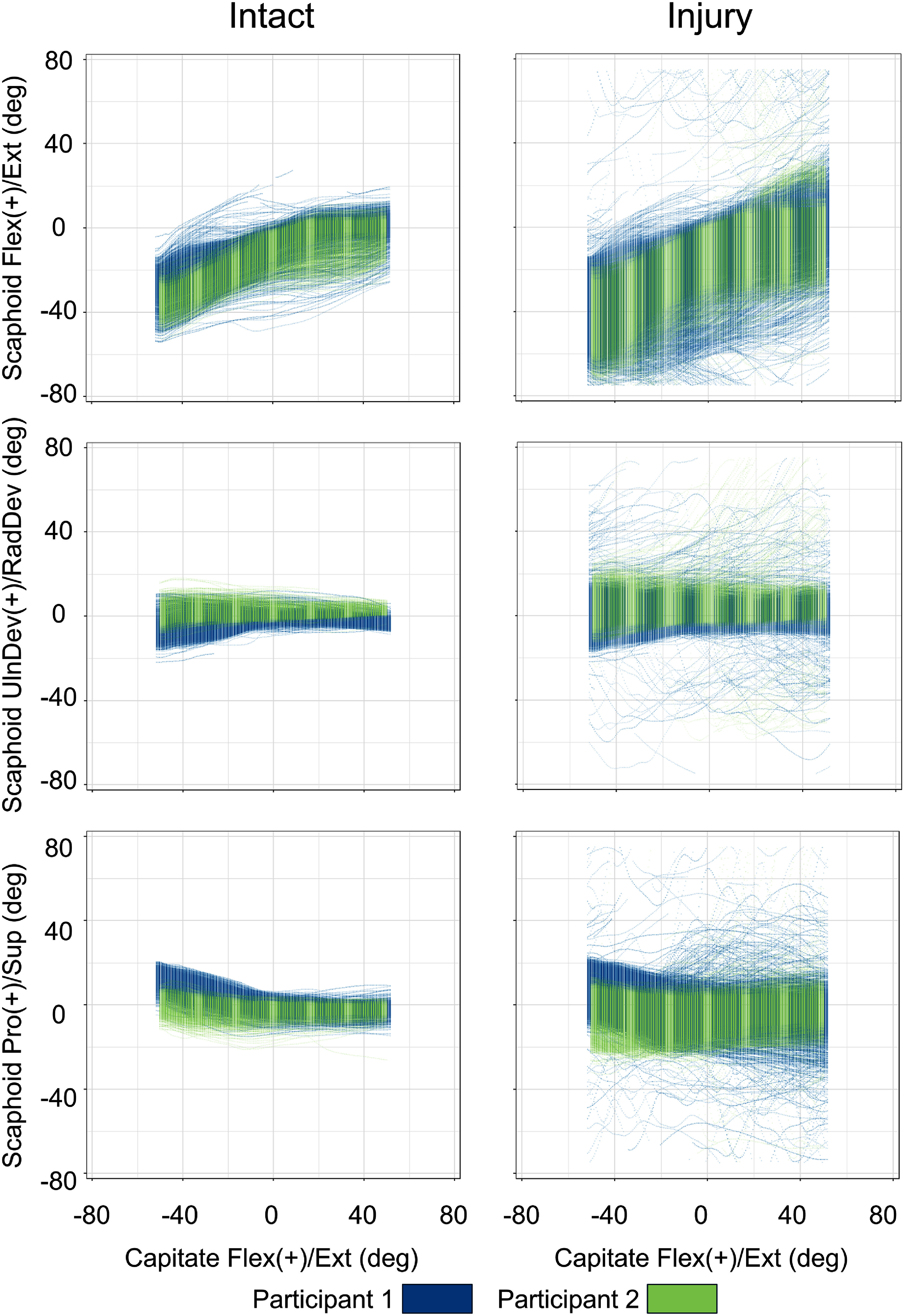
Scaphoid rotational kinematics from participant 1 and participants 2 during MC simulations of unresisted flexion-extension motion for models with all ligaments intact and those with some type of injury to the ligaments supporting the scapholunate joint. Simulations are shown as individual dots.

Material property variation created substantial variation in the radioscaphoid rotational kinematics angle across the range of flexion-extension motion (figure 9). The median scaphoid flexion-extension DOF increased by 30.8 deg. from maximum extension (50 deg. of capitate extension) to maximum flexion (50 deg. of capitate flexion). This was a greater magnitude of increase than those for the other rotational DOF, with the median scaphoid radial-ulnar deviation DOF increasing by 2.1 deg., and the median pronation-supination DOF decreasing by 11.6 deg. from maximum extension to maximum flexion. Additionally, the average total range between the 1st percentile and the 99th percentile for the scaphoid flexion-extension DOF across the range of motion was 31.2 deg. which was greater than those for the radial-ulnar deviation DOF of 15.1 deg. and the pronation-supination DOF of 16.8 deg. Between participant models, Participant 1 had increasing supination and ulnar deviation with increasing capitate extension while the values remained nearly constant for increasing capitate flexion. In contrast, Participant 2 models had more constant radial-ulnar deviation and pronation-supination DOFs for all capitate flexion-extension angles.

##### 3.3.1.2. Injured

Models that included some form of injury, in addition to the material property variation, resulted in increases in the range of scaphoid kinematics observed across the range of motion compared to intact simulations (figure 9). The median scaphoid flexion-extension DOF with injury had a greater magnitude of rotation across the flexion-extension motion than the intact models, increasing by 37.9 deg. from maximum extension (50 deg. of capitate extension) to maximum flexion (50 deg. of capitate flexion). Additionally, the average total range between the 1st percentile and the 99th percentile for the scaphoid flexion-extension DOF across the range of motion increased in the injured models to 52.8 deg. Similar increases in the total range were observed for the other DOF, with the average radial-ulnar deviation DOF range increasing to 21.1 deg. and the pronation-supination DOF increasing to 28.5 deg. Additionally, while the trends for the radial-ulnar deviation and pronation-supination DOFs appeared qualitatively similar to the intact predictions, the trends in scaphoid flexion-extension DOF appeared to have been more greatly altered with injury, particularly at extreme capitate flexion-extension angles.

#### 3.3.2. Radioscaphoid contact pressure

##### 3.3.2.1. Overall trends

**Figure 10.**
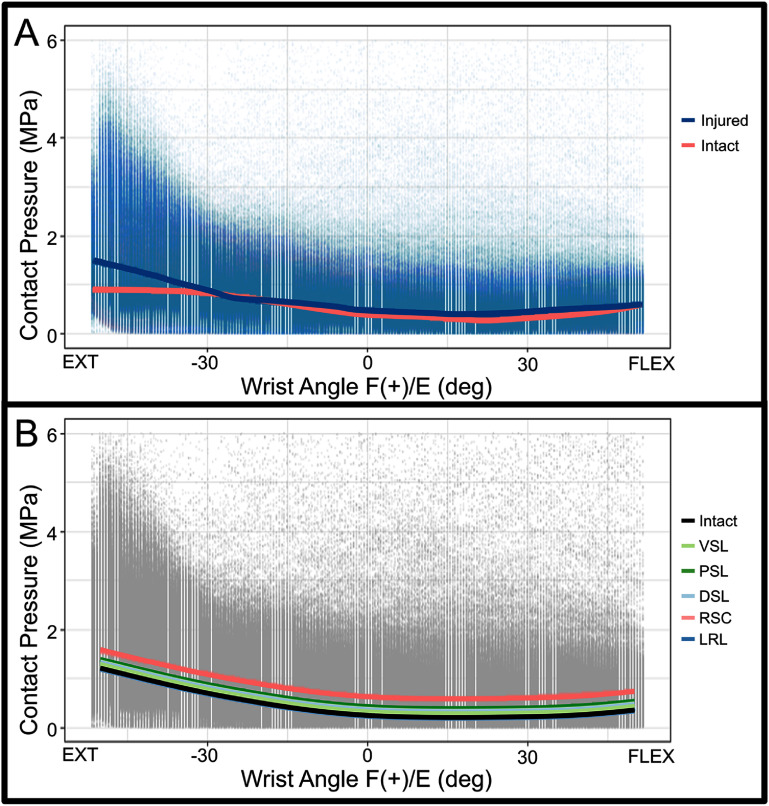
MC simulations predicting average radioscaphoid contact pressure as a function of wrist flexion-extension. (A) Solid lines are medians of pressure at each wrist angle for models without ligament injury (pink) and those with at least one ligament injury (blue).(B) GLME-estimated average radioscaphoid contact pressure following injury to the labeled ligament. In both graphs, individual simulations are shown as individual dots. The ligaments injured represent those at distinct levels of scapholunate interosseous ligament (SLIL) injuries, namely volar SLIL (VSL), proximal (membranous) SLIL (PSL), dorsal SLIL (DSL), radioscaphocapitate ligament (RSC), and the long radiolunate ligament (LRL).

The presence of SL ligament injuries had a noticeable impact on radioscaphoid contact pressure (figure 10(A)). While the presence of injury increased the radioscaphoid contact pressure across the range of flexion-extension, the effect was more pronounced at larger wrist extension angles (figure 10(A)). For angles between 50 deg. of flexion and 30 deg. of extension, the median of average contact pressures was within 0.16 MPa between models with and without injury. For extension angles beyond 30 degrees of extension, the median pressures differed, with the rate of increase in contact pressure of injured models being greater than that of the intact model (0.031 MPa/deg. and of 0.007 MPa/deg., respectively).

##### 3.3.2.2. GLME Results

The GLME results showed that the effect of injury to the different ligaments stabilizing the SL joint on radioscaphoid contact pressure is not the same (figure 10(B)). The RSC ligament was associated with a statistically significant 46.5% increase in radioscaphoid contact pressure relative to the average joint pressure independent of material properties, model, and injuries to other ligaments (table 5). In contrast, injury to the DSL was associated with only a 0.2% increase in the contact pressure, that was not statistically significant at (α = 0.001) (table 5).

**Table 5.** Generalized linear mixed effects (GLME) models were used to predict radioscaphoid contact pressure from ligament injury states in Participants 1 and 2. Values reported are coefficient estimates in true value (MPa) and normalized to average radioscaphoid contact pressure. Positive values represent increased pressure. Probabilistic metrics reported include standard error, t-statistic, and p value. * denotes statistical significance at *α* = 0.001. The ligaments injured represent those at distinct levels of scapholunate interosseous ligament (SLIL) injuries, namely volar SLIL (VSL), proximal (membranous) SLIL (PSL), dorsal SLIL (DSL), radioscaphocapitate ligament (RSC), and the long radiolunate ligament (LRL).

	Parameter	Estimate—true value (MPa)	Estimate -normalized to average	SE	*t*Stat	*p*Value
FE angle spline	Intercept	1.294	198.0%	0.006	232.01	<0.0001*
	Capitate angle DOF 1	−1.822	−278.8%	0.009	−213.35	<0.0001*
	Capitate angle DOF 2	−0.298	−45.5%	0.006	−52.19	<0.0001*

Ligament injury	VSL	0.003	0.4%	0.005	4.99	<0.0001*
	PSL	0.007	1.0%	0.005	13.33	<0.0001*
	DSL	0.001	0.2%	0.005	2.09	0.0363
	RSC	0.304	46.5%	0.005	59.46	<0.0001*
	LRL	−0.005	−0.7%	0.005	−8.99	<0.0001*

##### 3.3.2.3. Comparison to prior studies

**Figure 11.**
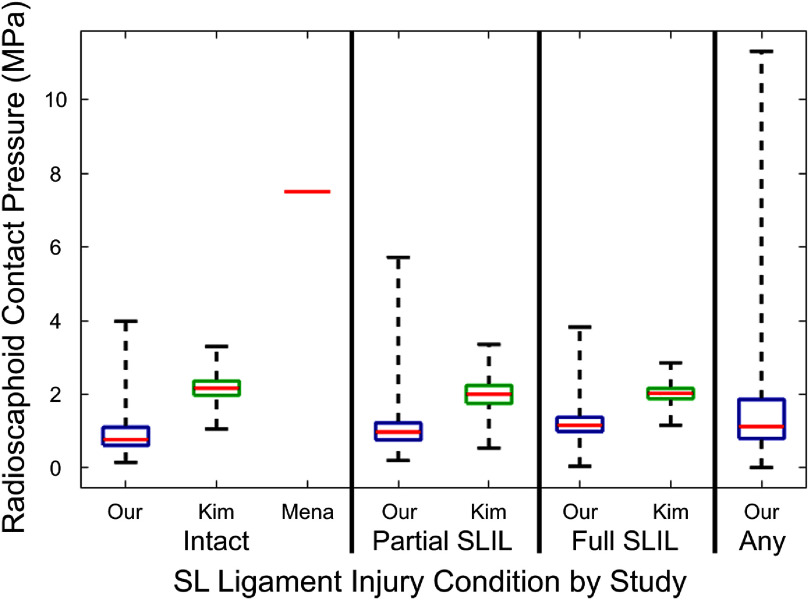
Distribution of radioscaphoid contact pressure from MC analysis at full extension for participant 1 and participant 2 compared to values reported in Kim *et al* and Mena *et al* (2024, 2025). ‘Intact’ refers to simulations with all ligaments intact. ‘Partial SLIL’ refers to simulations with at least one bundle of the scapholunate interosseous ligament (SLIL) injured with no extrinsic ligament injuries. ‘Full SLIL’ refers to simulations wherein all bundles of the SLIL are injured with no extrinsic ligament injury. ‘Any injury’ refers to models with at least one ligament injury (any combination of injury to the volar SLIL, proximal SLIL, dorsal SLIL, radioscaphocapitate, and long radiolunate).

When comparing the results herein with those for the radioscaphoid contact pressures in prior published work, the contact pressures differed (figure 11). While the median contact pressures observed herein were lower than those previously reported in Kim *et al* across all conditions, the range of contact pressures observed in that work was completely within the range of values observed herein for each ligament condition. Additionally, the total range of median radioscaphoid contact pressures in our model between intact, partially injured SLIL and fully injured SLIL, was 0.261 MPa; whereas Kim *et al* reported a range of 0.17 MPa. The values for the radioscaphoid contact pressure from Mena *et al* in an intact wrist were higher than the pressure observed herein, and in Kim *et al*, for the intact models. Across all conditions, the inclusion of any injury type (SLIL and extrinsic ligament injury) in our work resulted in the greatest contact pressures observed across all studies of approximately 11.50 MPa.

#### 3.3.3. Ligament forces

##### 3.3.3.1. Overall trends

**Figure 12.**
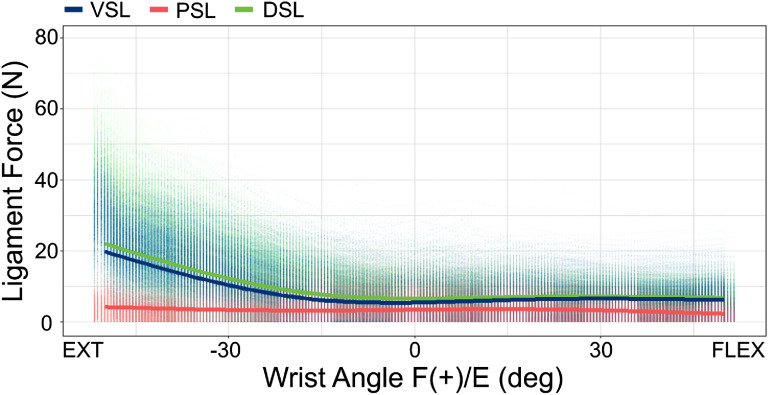
Simulated ligament forces during MC analysis in the scapholunate interosseous ligament (SLIL) bundles during wrist flexion and extension for participants 1 and 2 across all intact models. Solid lines are median ligament forces in SLIL bundles across all intact MC simulations. Points are individual MC simulations with all ligaments intact.

In all the intact models, the ligament forces in the SLIL bundles varied across the flexion-extension motion (figure 12). The ligament forces in the intact wrist increased in the DSL and VSL with increasing wrist extension angle, while force in the PSL remained nearly constant, changing by less than 2 N across the range of motion. (figure 12). The maximum ligament force observed at 50 deg. of wrist extension was greater in the DSL (79.4 N) compared to the VSL (61.1 N); however, the median forces at this position for all simulations were more similar, differing less than 6 N.

##### 3.3.3.2. GLME results

Using the GLME to account for material property variation, a statistically significant difference in average ligament force was found between the VSL and dorsal SLIL, with the VSL being of 1.6 N (table 6). Additionally, statistically significant differences were found between the DSL and PSL, with the average force in the DSL greater than that of the PSL by 25.7 N. Similar statistically significant differences were also found for the VSL compared to the PSL, where the force in the VSL was, on average, 24.1 N greater than that of the PSL.

**Table 6.** Generalized linear mixed effects (GLME) models for the ligament forces in the bundles of the scapholunate interosseous ligament (SLIL) from all MC simulations with all ligaments intact. The base case is dorsal SLIL (DSL); results represent differences from the base case for coefficients, the proximal SLIL (PSL) and the volar SLIL (VSL). Positive values represent an increase in ligament force. Probabilistic metrics are standard error, t-statistic, and p value. * denotes statistical significance at *α* = 0.001. The random effect is included since within-model results are not independent measures.

	Parameter	Estimate (N)	SE	*t*Stat	*p*Value
FE angle spline	Intercept	27.840	0.175	159.45	<0.0001*
	Capitate angle DOF 1	−42.580	0.167	−254.37	<0.0001*
	Capitate angle DOF 2	−6.613	0.111	−59.64	<0.0001*

Ligament	PSL	−25.650	0.020	−1303.91	<0.0001*
	VSL	−1.576	0.008	−202.76	<0.0001*
	Random scalar	0.009	0.011	0.86	0.3910
	Capitate angle DOF 1 proximal interaction	44.800	0.043	1039.25	<0.0001*
	Capitate angle DOF 2 proximal interaction	4.585	0.024	194.82	<0.0001*

## 4. Discussion

Computational modeling may enable new treatments for hand and wrist injuries, as it has for other joints, but is stifled by a lack of models of this anatomy. Most research to date includes a limited number of models confined to literature-derived average material properties, as their development and use have, up until now, been inefficient. A lack of variation in these model parameters increases the uncertainty in the results presented in these studies and decreases the utility of these models to answer clinically relevant questions. Improving this time limitation is crucial to increasing the personalization of these models and to increasing the adoption of FEM for hand and wrist research.

### 4.1. Workflow

We created a novel workflow to expedite the creation of personalized wrist models that run quickly and facilitate efficient material property variation. The workflow was entirely automated, combining non-linear morphing techniques with algorithmic predictions from open-source software tools, enabling the creation of new models in less than two hours. A major benefit of this workflow is its ability to estimate approximate soft tissue geometries based only on bone shapes. This makes it useful for distinct types of imaging, including static CT scans, as shown in this study. Additionally, the process uses advanced material models, like non-linear 1D connector fibers for ligaments (Blankevoort *et al* 1991, Yu *et al* 2001) and calibrated pressure overclosure materials (Halloran *et al* 2005, Huff *et al* 2020, Andreassen *et al* 2025a) for cartilage contact, which allows for rapid calculations. Model simulations can be completed on a standard desktop workstation in 45 s. A rapid development time, coupled with computationally efficient models, supports studies that require many simulations, such as Digital Twins and *in silico* clinical trials. Moreover, increased computational speed is key to clinical translation, as previous work has shown that the primary barrier to the clinical translation of computational modeling and simulation (CM&S) is the time needed for model development and usage (Lesage *et al* 2023). The process we developed in this study marks a crucial step towards wider use of models in orthopedics for the hand and wrist.

### 4.2. Model analyses

In addition to developing a novel workflow, we performed two analyses to highlight how this workflow can be used to create new models and to demonstrate potential applications, namely for developing personalized models and for MC analysis of soft-tissue injuries.

#### 4.2.1. Analysis 1

Analysis 1 calibrated the properties of ligaments to better align with an individual’s joint motion derived from 4DCT. Models of three asymptomatic individuals were developed using the created workflow, and ligament properties were calibrated to match the model predicted kinematics with those measured experimentally via 4DCT. The models successfully recreated complicated non-linear dynamics (figure 7) and improved the accuracy of predicting scaphoid and lunate kinematics in both the calibrated (unresisted) and unseen benchmark (resisted) trials across most DOF (tables 3 and 4), highlighting that calibration enhances model robustness in unseen scenarios. However, the improvements were greater in calibrated motions than in benchmark motions. Additionally, the reduction in RMSE varied between bones. Lastly, the improvement in RMSE was not uniform across models; Participant 2 exhibited the highest error rates, while Participants 1 and 3 generally had lower errors.

Taken together, these results emphasize the importance of including relevant structures and calibrating motions that can adequately stress the modeled ligaments. Participant 2 performed a gripping task that created minimal differences in kinematics across the motion cycle, providing few unique points to calibrate the kinematic changes in the scaphoid and lunate based on the overall wrist position. Consequently, there was a significant increase in the RMSE across most DOF between the uncalibrated motion (table 3) and the benchmark motion (table 4). In contrast, Participant 3 performed a flexion-extension task while Participant 1 engaged in a radial-ulnar deviation task, resulting in a broader range of wrist positions and larger ligament strain for accurately calibrating the ligaments across various joint angles. The ligament forces in figure 8 for Participant 3 demonstrate that the flexion-extension task resulted in a range of forces on the various ligaments throughout the motion cycle, including different forces for the same capitate flexion-extension angle resulting from subtle differences in the other DOF and direction of motion at that moment (figure 7). Thus, the RMSE between the uncalibrated motion (table 3) and benchmark motion (table 4) is much more similar between Participants 1 and 3 compared to the higher error observed in Participant 2. These results highlight the importance of developing robust models using a range of positions and situations to maximize their ability to predict unseen tasks.

#### 4.2.2. Analysis 2

In Analysis 2, models of two age- and sex- matched participants (Participant 1 and Participant 2) were used in a MC analysis to assess the impacts of ligament injury. The models showed a high sensitivity to model parameters with large ranges observed for kinematics, even with all ligaments intact (figure 9). Motions between the two models exhibited trends that were similar but still retained unique differences. When including all types of simulated SL ligament injuries, the overall median scaphoid FE shifted towards greater extension and the range of the bounds of simulations between the 1st and 99th percentile increased. These altered kinematics following injury created noticeable changes to the radioscaphoid contact pressure. While contact pressure remained nearly consistent with increasing extension (0.007 MPa/deg.), injuries caused median increases that were larger (0.031 MPa/deg.) emphasizing these soft-tissue injuries fundamentally impact long-term joint mechanics (figure 10(A)). The increasing radioscaphoid contact pressure at greater extension angles (figure 10(A)) followed similar patterns as the increasing ligament forces in the DSL and VSL in the intact limb (figure 12).

When injured, various SL ligaments increased the average radioscaphoid contact pressure (table 5 and figure 10(B)). Injury to the RSC caused a significant increase in radioscaphoid contact pressure even with all other ligaments remaining intact. In contrast, injury to the dorsal SLIL, considered the most crucial SL joint stabilizer (Berger 1996), caused no significant change in radioscaphoid contact pressure. The results of this work emphasize that certain metrics may be more directly associated with specific soft tissue structures. Further, this suggests that average changes to contact behavior may not be the most appropriate biomarker for wrist osteoarthritis progression.

### 4.3. Comparison to prior work

These findings generally agree with previous work. In Analysis 1, the increased accuracy of models calibrated to include personalized material properties, compared to those with average properties, has been demonstrated previously (Andreassen *et al* 2023, Razu *et al* 2023, Anantha Krishnan *et al* 2024). Still, differences in kinematic accuracies across the models (tables 3 and 4) suggest that calibration could be improved. Additionally, the poorer performance of the benchmark motion for Participant 2 highlights the importance of using dynamically changing motion that reaches extremes to maximize the potential of models to make robust predictions to unseen scenarios. Certain joint motions better inform specific ligament behaviors and, depending on the intended model use, are important to achieve adequate accuracy. In Analysis 2, intact radioscaphoid contact pressures overlap those reported in Kim *et al* (2024) but not Mena *et al* (2025); however, average values were less than reported elsewhere (figure 11). Radioscaphoid pressures were also similar to Kim *et al* for the intact, partial, and fully injured SLIL but ranges observed herein were larger. Additionally, the ligament forces observed in the MC analysis agree with prior cadaveric testing, which showed that maximum SLIL force was observed at maximum extension, with an average total SLIL force of 20.0 ± 3.3 N at 30° extension (Dimitris *et al* 2015). Our models similarly predicted the highest loads with extension, with an average total SLIL force of 26.0 N at 30° extension (figure 11).

### 4.4. Limitations

This work is not free of limitations. First, only three carpal bones were included. Other models often include all carpal bones and more ligaments. Some differences in kinematics likely resulted from the absence of certain bones and ligaments. In Analysis 1, the improvements in the scaphoid were superior to the lunate, and kinematics for some parts of the time domain could not be recreated by the model (figure 7). These differences may be improved with the inclusion of additional structures. In Analysis 2, including additional ligaments could change patterns and interpretation. We chose to use a limited set of bones, and consequentially, ligaments based on availability of their kinematics from our prior work (Trentadue *et al* 2024b). Previous studies have used the same subset of bones to develop a model of radius contact pressures and ligament stress in the SLIL (Marques *et al* 2021). Crucially, the workflow we have developed is not unique to these bones and could be applied more broadly.

Another limitation is the narrow scope of the validation. In Analysis 1, the benchmark trials were used to validate the accuracy of the models during unseen kinematics. While this mimics validation for other joints (Erdemir *et al* 2019), we were unable to directly validate the contact pressures and ligament loads, as there is no practical method to measure these *in vivo*. Still, in Analysis 2, the model predictions for both contact pressure (Mena *et al* 2025, Kim *et al* 2024) and ligament loads are consistent with existing work (Dimitris *et al* 2015).

Another limitation is the comparison of radioscaphoid contact pressures to those reported in the literature. Previous studies have reported maximum contact pressure at a single node while this work reported average contact pressure (Kim *et al* 2024, Mena *et al* 2025). We chose to use the average pressure, as it is less affected by surface reconstruction error, mesh discretization, and choice of contact model than maximum pressure (Wachman *et al* 2016, Dassault Systèmes 2021).

Last, this study has a relatively small sample size, insufficient to make broad claims. However, the goal of this work was first to develop a workflow that would demonstrate superior efficiency for model development and use over existing work in the wrist. The investigations performed were meant to highlight some potential uses of these improved models relating to ongoing topics related to Digital Twins and *in silico* clinical trials, as opposed to comprehensively evaluating wrist behavior. However, future work will aim to use the workflow developed herein to perform each analysis using more individuals, including those with pathology, to better answer clinically relevant questions.

### 4.5. Clinical relevance

#### 4.5.1. Current

The results found herein provide additional information beyond what has historically been possible from benchtop or human participant testing alone, which may be relevant to clinical contexts. Differences in ligament forces in the intact model were often statistically significant but not necessarily by an amount that would be physically meaningful; for instance, in Analysis 2, ligament forces in the VSL versus DSL differed by only 1.6 N (table 6). While this difference is not likely clinically relevant by itself, it may explain why the VSL is frequently injured before the DSL: although the forces are nearly identical, the failure strength of the VSL is significantly lower (Berger *et al* 1999). Additionally, the individual motions between participants may result in differing kinematics where forces in the VSL exceed those of the DSL, such as in Participant 3 (figure 8). These models provide evidence to potentially explain the general progression of SLIL injury from VSL to DSL in most cases (Zhou *et al* 2024). While the sample size is small, the consistent patterns for the VSL and the dorsal radiocarpal (DRC) ligament across all models in Analysis 1 suggest that some ligaments may be consistently under residual stress (VSL) in a neutral wrist position, while others are slack (DRC). This has implications for clinical practice, as surgeries involving these ligaments may need to account for not only the stiffness of the original ligaments but also their resting tension, something that has been underreported in the literature in the hand and wrist, to date. Lastly, the changing radioscaphoid contact pressures following injury show how soft-tissue injuries can alter joint motion and may help explain how soft-tissue injuries impact contact mechanics in the short term, which could be relevant to the development of joint osteoarthritis in the long-term (Watson and Ballet 1984). Still, results should be interpreted in the context of experimental conditions and clinical experience.

#### 4.5.2. Future

In addition to these relevant clinical takeaways, the analyses used herein highlight how these models can be used to advance areas of established research importance that have high potential for clinical translation, namely, Digital Twins and *in silico* clinical trials.

Digital twins are considered an important part of future personalized medicine in areas like arthroscopic surgery (Bjelland *et al* 2022), fracture repair (Andres *et al* 2025), and joint replacement (Dean *et al* 2024), and fundamentally work by accurately representing individual patients (Viceconti *et al* 2023). The workflow created enables the rapid creation of personalized models without needing to identify soft-tissue structures individually. Analysis 1 showed a way to improve the model’s material properties for better personalization, using measurements from each person’s motion captured by 4DCT. Once these models are created, they can measure a range of factors that are impractical, or impossible to obtain through other methods, such as ligament forces. These ligament forces may offer clinicians new ways to identify injuries. Additionally, by accurately modeling the underlying mechanics of the hand, these models can be used to explore different injury scenarios and repair options, aiding in the identification of targets for restoring function and assessing different surgical methods. The calibrated ligament properties can be used to help the choice of grafts or materials for ligament repairs to better match the individual’s native material property. Finally, Analysis 2 demonstrates how these models can identify short-term changes in joint mechanics due to soft-tissue injuries that may impact long-term prognosis. Understanding these changes is crucial for assessing the effects of injuries on patients and can inform clinicians about the best timing for surgery to prevent long-term issues, such as increased pressure on the joint and greater cartilage wear.

In addition to Digital Twins, these models, following new guidelines from the FDA and ASME, can be used in virtual clinical trials (ASME 2018, FDA 2023). As shown in Analysis 2, a key benefit of these models is their ability to be slightly adjusted to better understand different outcomes under uncertain conditions. Unlike traditional clinical trials, which only include enrolled participants, these models can create synthetic individuals with minor changes, increasing the number of samples compared to earlier studies. With more simulations, MC Analysis can find connections between different factors that may affect important results while considering natural physiological differences, which is often difficult with current methods. Analysis 2 demonstrated that the models could identify links between changes in contact pressure due to various ligament injuries, even while accounting for the complex relationships between ligament injuries and the natural differences in material properties among people. Additionally, these models can simulate individuals with rare combinations of features and in extreme situations. Including these individuals in traditional clinical trials can be difficult, but it is essential for testing the effectiveness of any medical device. Figure 9 illustrates how these models can predict performance of most individuals (between the 1st and 99th percentiles) and shows extra simulations of extreme cases outside these general bounds. Identifying these cases is important because developed clinical solutions may not be suitable for them. This can lead to the development of new alternative procedures or device variants that aim to improve the performance of interventions for these difficult to treat cases.

## 5. Conclusion

We have developed a novel automated workflow to rapidly create personalized wrist models that have efficient computational speeds. The workflow was used to develop three personalized computational models of the wrist from three living participants and created models with computational speeds exceeding those of prior work. These models were then used in two analyses to demonstrate the relevance of rapid models by enabling variation of internal parameters, namely ligament properties, to investigate the impacts of material variation and injury.

The key findings of this study were the following: (1) Combining template models with novel morphing and algorithmic techniques improves the time required to develop new personalized models and enables model creation from imaging modalities that traditionally lack soft-tissue discernment. (2) Calibration of wrist ligament material properties by matching model predicted kinematics to experimentally obtained kinematics improves model predictions even in unseen loaded benchmark data but improves further when calibrating using data where ligaments were sufficiently engaged. (3) Monte Carlo based variation of ligament material properties and ligament injuries enables investigation of the interplay between soft-tissue injuries and highlighted that the injuries to the scapholunate interosseous ligament (SLIL) impacts radioscaphoid contact pressure.

These efforts improve our ability to model unique patients and demonstrate a path towards patient-specific models for developing digital twins, pre-surgical planning tools, and *in silico* clinical trials. Moreover, this work provides a path to better understanding of patient-specific differences by creating personalized computational models that will improve our understanding of the short- and long-term effects of injury and may guide surgical treatment. Overall, we present a pipeline towards efficient personalized wrist modeling that enables new analyses for improved understanding of hand and wrist injuries. The original raw data, working model for participant 1, and implementation of the model development code have been made publicly available.

## Data Availability

Following IRB approval from (IRB# 25-012254), various raw data, models, and code to perform the workflow created have been made publicly available online. The models, data, and software created are available online on the public repository, Zenodo. The raw data includes geometries and kinematics of the bones for the participants captured with the four-dimensional computed tomography and is available at: https://doi.org/10.5281/zenodo.18166548 (Andreassen *et al* 2026a). The working personalized Abaqus model for Participant 1 with the calibrated ligament material properties is available at: https://doi.org/10.5281/zenodo.18166610. The MATLAB code and software used to implement the model workflow is available at: https://doi.org/10.5281/zenodo.18166628 (Andreassen *et al* 2026b). Model Creation & Verification available at https://doi.org/10.1088/1873-4030/ae8542/data1. Graphical Abstract available at https://doi.org/10.1088/1873-4030/ae8542/data2.

## Credit

**TEA:** Conceptualization, Formal analysis, Investigation, Methodology, Software, Visualization, Writing -Original Draft Preparation **TPT:** Investigation, Validation, Data Curation, Writing—Original Draft Preparation **ART:** Conceptualization, Validation, Data Curation, Resources, Project Administration **KA:** Conceptualization, Validation, Supervision **SK:** Validation, Supervision **KDZ:** Conceptualization, Validation, Funding Acquisition, Resources, Supervision. All authors performed Writing -Review and Editing.

## References

[mepae8542bib1] Alonso Rasgado T, Zhang Q, Jimenez Cruz D, Bailey C, Pinder E, Mandaleson A (2017). Analysis of tenodesis techniques for treatment of scapholunate instability using the finite element method. Int. J. Num. Method Biomed. Eng..

[mepae8542bib2] Anantha Krishnan A, Myers C A, Scinto M, Marshall B N, Clary C W (2024). Specimen-specific finite element representations of implanted hip capsules. Comput. Methods Biomech. Biomed. Eng..

[mepae8542bib3] Anderson A E, Ellis B J, Weiss J A (2007). Verification, validation and sensitivity studies in computational biomechanics. Comput. Methods Biomech. Biomed. Eng..

[mepae8542bib4] Andersson J K, Garcia-elias M (2013). Dorsal scapholunate ligament injury: a classification of clinical forms. J. Hand Surg. Eur..

[mepae8542bib5] Andreassen T E, Hume D R, Hamilton L D, Hegg S L, Higinbotham S E, Shelburne K B (2025a). Validating subject-specific knee models from *in vivo* measurements. Front. Bioeng. Biotechnol..

[mepae8542bib6] Andreassen T E, Hume D R, Hamilton L D, Higinbotham S E, Shelburne K B (2024). Automated 2D and 3D finite element overclosure adjustment and mesh morphing using generalized regression neural networks. Med. Eng. Phys..

[mepae8542bib7] Andreassen T E, Laz P J, Erdemir A, Besier T F, Halloran J P, Imhauser C W (2023). Deciphering the ‘Art’ in modeling and simulation of the knee joint: assessing model calibration workflows and outcomes. J. Biomech. Eng..

[mepae8542bib8] Andreassen T E, Trentadue T P, Thoreson A R, An K N, Kakar S, Zhao K D (2025b). Rapid development of efficient participant-specific computational models of the wrist.

[mepae8542bib9] Andreassen T E, Trentadue T P, Thoreson A R, An K, Kakar S, Zhao K D (2026a). Rapid personalized computational modeling of the wrist - data (Zenodo).

[mepae8542bib10] Andreassen T E, Trentadue T P, Thoreson A R, An K, Kakar S, Zhao K D (2026b). Rapid personalized computational modeling of the wrist - data (Zenodo).

[mepae8542bib11] Andres A (2025). advantages of digital twin technology in orthopedic trauma surgery—exploring different clinical use cases. Sci. Rep..

[mepae8542bib12] Apergis E (2013). Wrist Anatomy. Fracture-Dislocations of the Wrist.

[mepae8542bib13] ASME (2018). Assessing Credibility of Computational Modeling through Verification and Validation: Application to Medical Devices.

[mepae8542bib14] Bajuri M N, Abdul Kadir M R, Murali M R, Kamarul T (2013). Biomechanical analysis of the wrist arthroplasty in rheumatoid arthritis: a finite element analysis. Med. Biol. Eng. Comput..

[mepae8542bib15] Baldwin M A, Clary C W, Fitzpatrick C K, Deacy J S, Maletsky L P, Rullkoetter P J (2012). Dynamic finite element knee simulation for evaluation of knee replacement mechanics. J. Biomech..

[mepae8542bib16] Baldwin M A, Laz P J, Stowe J Q, Rullkoetter P J (2009). Efficient probabilistic representation of tibiofemoral soft tissue constraint. Comput. Methods Biomech. Biomed. Eng..

[mepae8542bib17] Barr A E, Barbe M F, Clark B D (2004). Work-related musculoskeletal disorders of the hand and wrist: epidemiology, pathophysiology, and sensorimotor changes. J. Orthop. Sports Phys. Ther..

[mepae8542bib18] Berger R A (1996). The gross and histologic anatomy of the scapholunate interosseous ligament. J. Hand Surg. Am..

[mepae8542bib19] Berger R A (2001). The anatomy of the ligaments of the wrist and distal radioulnar joints. Clin. Orthop. Relat. Res..

[mepae8542bib20] Berger R A, Imeada T, Berglund L, An K-N (1999). Constraint and material properties of the subregions of the scapholunate interosseous ligament. J. Hand Surg. Am..

[mepae8542bib21] Bjelland O, Rasheed B, Schaathun H G, Pedersen M D, Steinert M, Hellevik A I, Bye R T (2022). Toward a digital twin for arthroscopic knee surgery: a systematic review. IEEE Access.

[mepae8542bib22] Blankevoort L, Kuiper J H, Huiskes R, Grootenboer H J (1991). Articular contact in a three-dimensional model of the knee. J. Biomech..

[mepae8542bib23] Butler D L, Kay M D, Stouffer D C (1986). Comparison of material properties in fascicle-bone units from human patellar tendon and knee ligaments. J. Biomech..

[mepae8542bib24] Chang N, Suh N (2022). Anatomy and biomechanics of scapholunate ligament. Arthroscopy and Endoscopy of the Elbow, Wrist and Hand.

[mepae8542bib25] Coburn J C, Upal M A, Crisco J J (2007). Coordinate systems for the carpal bones of the wrist. J. Biomech..

[mepae8542bib26] Dassault Systèmes Simulia Corp (2021). ABAQUS Analysis User’s Manual.

[mepae8542bib27] de Putter C E, Selles R W, Polinder S, Panneman M J, Hovius S E, van Beeck E F (2012). Economic impact of hand and wrist injuries: health-care costs and productivity costs in a population-based study. J. Bone Joint Surg. Am..

[mepae8542bib28] Dean M C, Oeding J F, Diniz P, Seil R, Samuelsson K, Group E A I W (2024). Leveraging digital twins for improved orthopaedic evaluation and treatment. J. Exp. Orthop..

[mepae8542bib29] Dimitris C, Werner F W, Joyce D A, Harley B J (2015). Force in the scapholunate interosseous ligament during active wrist motion. J. Hand Surg. Am..

[mepae8542bib30] Erdemir A, Besier T F, Halloran J P, Imhauser C W, Laz P J, Morrison T M (2019). Deciphering the ‘Art’ in modeling and simulation of the knee joint: overall strategy. J. Biomech. Eng..

[mepae8542bib31] Favre P, Maquer G, Henderson A, Hertig D, Ciric D, Bischoff J E (2021). In silico clinical trials in the orthopedic device industry: from fantasy to reality?. Ann. Biomed. Eng..

[mepae8542bib32] Gislason M K, Stansfield B, Nash D H (2010). Finite element model creation and stability considerations of complex biological articulation: the human wrist joint. Med. Eng. Phys..

[mepae8542bib33] Guo X, Fan Y, Li Z-M (2009). Effects of dividing the transverse carpal ligament on the mechanical behavior of the carpal bones under axial compressive load: a finite element study. Med. Eng. Phys..

[mepae8542bib34] Halloran J P, Petrella A J, Rullkoetter P J (2005). Explicit finite element modeling of total knee replacement mechanics. J. Biomech..

[mepae8542bib35] Hamilton L D, Andreassen T E, Myers C, Shelburne K B, Clary C, Rullkoetter P J (2022). Supine leg press as an alternative to standing lunge in high-speed stereo radiography. J. Biomech..

[mepae8542bib36] Heller M O (2022). Chapter 32 - Finite element analysis in orthopedic biomechanics. Human Orthop. Biomech..

[mepae8542bib37] Huff D N, Myers C A, Rullkoetter P J (2020). Impact of alignment and kinematic variation on resistive moment and dislocation propensity for THA with lipped and neutral liners. Biomech. Model. Mechanobiol..

[mepae8542bib38] Hume D R, Rullkoetter P J, Shelburne K B (2020). ReadySim: a computational framework for building explicit finite element musculoskeletal simulations directly from motion laboratory data. Int. J. Num. Methods Biomed. Eng..

[mepae8542bib39] Kim S, Nyaaba W, Mzeihem M, Mejia A, Gonzalez M, Amirouche F (2024). Wrist kinetics post-scapholunate dissociation: experimental and computational analysis of scapholunate interosseous ligament injury. J. Hand Surg. Glob. Online.

[mepae8542bib40] Leonardo-diaz R, Alonso-rasgado T, Jimenez-cruz D, Bailey C G, Talwalkar S (2020). Performance evaluation of surgical techniques for treatment of scapholunate instability in a type II wrist. Int. J. Num. Method Biomed. Eng..

[mepae8542bib41] Lesage R, Van Oudheusden M, Schievano S, Van Hoyweghen I, Geris L, Capelli C (2023). Mapping the use of computational modelling and simulation in clinics: a survey. Front. Med. Technol..

[mepae8542bib42] Li Z, Kim J-E, Davidson J S, Etheridge B S, Alonso J E, Eberhardt A W (2007). Biomechanical response of the pubic symphysis in lateral pelvic impacts: a finite element study. J. Biomech..

[mepae8542bib43] Maas S A, Ellis B J, Ateshian G A, Weiss J A (2012). FEBio: finite elements for biomechanics. ASME J. Biomech. Eng..

[mepae8542bib44] Malbouby V, Gibbons K D, Bursa N, Ivy A K, Fitzpatrick C K (2025). Efficient development of subject-specific finite element knee models: automated identification of soft-tissue attachments. J. Biomech..

[mepae8542bib45] Marai G E, Crisco J J, Laidlaw D H (2006). A kinematics-based method for generating cartilage maps and deformations in the multi-articulating wrist joint from CT images.

[mepae8542bib46] Marques R, Melchor J, Sanchez-montesinos I, Roda O, Rus G, Hernandez-cortes P (2021). Biomechanical finite element method model of the proximal carpal row and experimental validation. Front. Physiol..

[mepae8542bib47] Mena A, Wollstein R, Baus J, Yang J (2023). Finite element modeling of the human wrist: a review. J. Wrist Surg..

[mepae8542bib48] Mena A, Wollstein R, Yang J (2025). Development of a finite element model of the human wrist joint with radial and ulnar axial force distribution and radiocarpal contact validation. J. Biomech. Eng..

[mepae8542bib49] Mizuseki T, Ikuta Y (1989). The dorsal carpal ligaments: their anatomy and function. J. Hand Surg. Br..

[mepae8542bib50] Moerman K M (2018). GIBBON: the geometry and image-based bioengineering add-on. J. Open Source Softw..

[mepae8542bib51] Moritomo H (2013). Anatomy and clinical relevance of the ulnocarpal ligament. J. Wrist Surg..

[mepae8542bib52] Nagao S, Patterson R M, Buford W L, Andersen C R, Shah M A, Viegas S F (2005). Three-dimensional description of ligamentous attachments around the lunate. J. Hand Surg. Am..

[mepae8542bib53] Nikolopoulos F V, Apergis E P, Poulilios A D, Papagelopoulos P J, Zoubos A V, Kefalas V A (2011). Biomechanical properties of the scapholunate ligament and the importance of its portions in the capitate intrusion injury. Clin. Biomech..

[mepae8542bib54] Pillet H, Bergamini E, Rochcongar G, Camomilla V, Thoreux P, Rouch P, Cappozzo A, Skalli W (2016). Femur, tibia and fibula bone templates to estimate subject-specific knee ligament attachment site locations. J. Biomech..

[mepae8542bib55] Razu S S, Jahandar H, Zhu A, Berube E E, Manzi J E, Pearle A D (2023). Bayesian calibration of computational knee models to estimate subject-specific ligament properties, tibiofemoral kinematics, and anterior cruciate ligament force with uncertainty quantification. J. Biomech. Eng..

[mepae8542bib56] Ringler M D (2013). MRI of wrist ligaments. J. Hand Surg. Am..

[mepae8542bib57] Rooks N B, Schneider M T Y, Erdemir A, Halloran J P, Laz P J, Shelburne K B (2021). Deciphering the ‘art’ in modeling and simulation of the knee joint: variations in model development. J. Biomech. Eng..

[mepae8542bib58] Trentadue T P (2023a). Assessing carpal kinematics following scapholunate interosseous ligament injury *ex vivo* using four-dimensional dynamic computed tomography. Clin. Biomech..

[mepae8542bib59] Trentadue T P (2024b). Detection of scapholunate interosseous ligament injury using dynamic computed tomography-derived arthrokinematics: a prospective clinical trial. Med. Eng. Phys..

[mepae8542bib60] Trentadue T P, Lopez C, Breighner R E, Fautsch K, Leng S, Holmes Iii D R, Moran S L, Thoreson A R, Kakar S, Zhao K D (2023b). Evaluation of scapholunate injury and repair with dynamic (4D) CT: a preliminary report of two cases. J. Wrist Surg..

[mepae8542bib61] Trentadue T P, Thoreson A, Lopez C, Breighner R E, Leng S, Holmes D R, Kakar S, Rizzo M, Zhao K D (2024a). Morphology of the scaphotrapeziotrapezoid joint: a multi-domain statistical shape modeling approach. J. Orthop. Res..

[mepae8542bib62] Trentadue T P, Thoreson A, Lopez C, Breighner R E, Leng S, Kakar S, Rizzo M, Zhao K D (2025). Sex differences in photon-counting detector computed tomography-derived scaphotrapeziotrapezoid joint morphometrics. Skeletal Radiol..

[mepae8542bib63] U.S. Food and Drug Administration (2023). Assessing the credibility of computational modeling and simulation in medical device submissions: guidance for industry and food and drug administration staff. https://www.fda.gov/regulatory-information/search-fda-guidance-documents/assessing-credibility-computational-modeling-and-simulation-medical-device-submissions.

[mepae8542bib64] Varga P, Schefzig P, Unger E, Mayr W, Zysset P K, Erhart J (2013). Finite element based estimation of contact areas and pressures of the human scaphoid in various functional positions of the hand. J. Biomech..

[mepae8542bib65] Viceconti M, De Vos M, Mellone S, Geris L (2023). Position paper from the digital twins in healthcare to the Virtual Human Twin: a moon-shot project for digital health research. IEEE J. Biomed. Health Inform..

[mepae8542bib66] Wachman K, Saputra E, Ismail R, Jamari J, Bayuseno A P (2016). Analysis of the contact area of smooth and rough surfaces in contact with sphere indenter using finite element method.

[mepae8542bib67] Walker-bone K, Palmer K T, Reading I, Coggon D, Cooper C (2004). Prevalence and impact of musculoskeletal disorders of the upper limb in the general population. Arthritis Rheum.

[mepae8542bib68] Wang W L, Abboudi J, Gallant G, Jones C, Kirkpatrick W, Kwok M, Liss F, Takei T R, Wang M, Ilyas A M (2022). Radiographic incidence and functional outcomes of distal radius fractures undergoing volar plate fixation with concomitant scapholunate widening: a prospective analysis. Hand.

[mepae8542bib69] Ward P J, Fowler J R (2015). Scapholunate ligament tears: acute reconstructive options. Orthop. Clin. North Am..

[mepae8542bib70] Watson H K, Ballet F L (1984). The SLAC wrist: scapholunate advanced collapse pattern of degenerative arthritis. J. Hand Surg. Am..

[mepae8542bib71] Weiss J A, Gardiner J C (2001). Computational modeling of ligament mechanics. Crit Rev. Biomed. Eng..

[mepae8542bib72] Yu C H, Walker P S, Dewar M E (2001). The effect of design variables of condylar total knees on the joint forces in step climbing based on a computer model. J. Biomech..

[mepae8542bib73] Zhang J, Yao X, Song Y, Yin P (2024). Establishment and preliminary evaluation of CT-based classification for distal radius fracture. Sci. Rep..

[mepae8542bib74] Zhang Q, Adam N C, Hosseini Nasab S H, Taylor W R, Smith C R (2021). Techniques for *in vivo* measurement of ligament and tendon strain: a review. Ann. Biomed. Eng..

[mepae8542bib75] Zhao K, Breighner R, Holmes D, Leng S, McCollough C, An K-N (2015). A technique for quantifying wrist motion using four-dimensional computed tomography: approach and validation. J. Biomech. Eng..

[mepae8542bib76] Zhou J Y, Jodah R, Joseph L P, Yao J (2024). Scapholunate ligament injuries. J. Hand Surg. Glob. Online.

